# Drive-By Time-Varying Feature Extraction for Bridge Damage Detection Using Second-Order Synchrosqueezing Transform

**DOI:** 10.3390/s26165170

**Published:** 2026-08-15

**Authors:** Mingzhe Gao, Xinqun Zhu, Jianchun Li

**Affiliations:** School of Civil and Environmental Engineering, University of Technology Sydney, Ultimo, NSW 2007, Australia; mingzhe.gao@alumni.uts.edu.au (M.G.); jianchun.li@uts.edu.au (J.L.)

**Keywords:** time–frequency characteristics, vehicle–bridge interaction, second-order synchrosqueezing transform, bridge structural health monitoring, time–frequency representation, instantaneous frequency

## Abstract

Recently, drive-by bridge structural health monitoring has gained increasing attention due to its potential to be a cost-effective way to monitor the highway infrastructure. The pre-installed sensory system on a passing vehicle is used to capture the spatiotemporal response of the bridge for structural health monitoring. The vehicle passing over the bridge is a time-varying process, and it is a big challenge to extract the time-varying characteristics of vehicle–bridge interaction systems for structural health monitoring. This paper aims to develop a drive-by time-varying feature extraction approach for bridge structural damage detection using the second-order synchrosqueezing transform. The research first examined the impact of various factors on the frequency changes in VBI systems, including the vehicle mass, stiffness, speed, road surface profiles, measurement noise, and different types of damage. When compared with traditional synchrosqueezing transform, the proposed method provides a clearer and more accurate time–frequency representation. A 6 m-long two-span bridge model is also built in the laboratory and the pre-installed wireless sensory system on a passing vehicle captures the vehicle and bridge interaction response. The time-varying features are extracted from dynamic responses of the vehicle passing over the bridge using the proposed method. Numerical and experimental results show that the proposed approach is effective and accurate enough to extract the time-varying features for bridge damage detection.

## 1. Introduction

Bridge structures continuously deteriorate due to ageing, and environmental and operational loading. It is essential to detect bridge damage at early stage for structural safety and provide quantitative data for cost-effective infrastructure asset management. Traditional bridge health monitoring involves the direct installation of sensors on the bridge, and the damage features are extracted from the bridge vibration [1]. Deploying numerous sensors remains a key challenge in bridge health monitoring, especially for large volume of small–medium-span bridges with limited budgets. Also, the variabilities of the field environments, especially the traffic conditions significantly impede reliable bridge health monitoring. Recently, the inclusion of the vehicle–bridge interaction (VBI) information for bridge structural damage identification has attracted great attention of researchers and practical engineers [2].

The vehicle passing over the bridge induces complex vehicle–bridge dynamic interaction. Yang and Lin [3] presented analytical solutions of a simplified VBI system with a small vehicle/bridge mass ratio. The bridge dynamic response is governed by the vehicle driving frequency and bridge frequency components. The vehicle response is governed by the driving frequencies, and vehicle and bridge frequencies with shifts. An et al. [4] presented a numerical analysis of dynamic responses for VBI systems with long-span continuous girder bridges. Tan et al. [5] analysed dynamic responses of the vehicle–bridge coupled system to evaluate the reinforcement effect on dynamic behaviour of an arch bridge. The vehicle passing over the bridge basically generates a wealth of data containing damage-sensitive features for structural condition assessment. Zhu and Law [6] presented an overview of inverse problems in VBI dynamics. Structural parameter identification with the moving vehicle as excitation of the bridge structure is one kind of inverse problems in VBI systems.

The identification of time-varying characteristics from dynamic responses of the bridge under moving vehicles is a challenge task. Yang et al. [7] firstly presented analytical solutions of VBI system and discussed frequency variation patterns with special cases of moving mass and moving load. The vehicle parameters have significant effect on the frequency shifts [8,9]. Li et al. [10] proposed a method to extract time-varying of bridges under the passage of vehicles using synchroextracting transform. An improved empirical wavelet transform along with edge detection was also proposed to estimate the instantaneous frequencies (IFs) of the bridge [11]. Singh and Sadhu [12] presented a hybrid method by integrating time-varying filtering-based empirical mode decomposition with synchroextracting transform to track IFs of the bridge under moving vehicles. Zhang et al. [13] proposed a modified S-transform reassignment technique to extract time-varying characteristics of VBI systems from bridge dynamic responses. A modified variational nonlinear chirp mode decomposition was proposed to extract IFs from dynamic responses of the bridge under moving vehicles [14]. The vehicle passing over the bridge is a time-varying process and different methods have been developed to extract time-varying characteristics of the bridge under moving vehicles. There is a little study on bridge damage detection using these time-varying features. Recently, the damage index from the IFs of bridges was calculated to assess the bridge damage [15]. As above, the sensors are directly installed on the bridge to capture dynamic responses of the bridge under moving vehicles, and it makes costly and labour-intensive for practical engineering applications.

Indirect bridge health monitoring, also known as the drive-by method or vehicle scanning method, has gained significant interest from researchers recently due to its low cost of deployment, management and maintenance [16]. Yang et al. [17] proposed this idea of indirect approach to extract modal parameters of bridge structures from dynamic responses of a passing vehicle. The spatial–temporal response of the bridge is captured by a passing vehicle with pre-installed sensors and then the bridge structural damage is identified using dynamic response sensitivity analysis [18]. A literature of this topic to extract bridge frequencies from a passing vehicle pre-installed with vibration sensors is presented in the textbook by Yang et al. [19]. Recently, smartphones in a large number of moving vehicles are used as mobile sensors to extract modal parameters from everyday vehicle trip data [20]. To improve the frequency resolution due to the short-time monitoring record of the vehicle passing over the bridge, in-fleet monitoring using connected and autonomous vehicles is introduced by Shokravi et al. [21]. However, the bridge damage is a local phenomenon, and bridge modal parameters are insensitive to the damage.

The VBI system is naturally time-varying. The vehicle trespassing the damage location excites and captures the local response, which is much more sensitive to the damage. It is essential to explore time-varying characteristics of the VBI system for bridge damage detection. Short-time Fourier transform (STFT) and continuous wavelet transform (CWT) are two most typical techniques to obtain a two-dimensional time–frequency representation (TFR) of non-stationary signals [22,23]. STFT’s advantage lies in its ability to provide better time localization in high-frequency regions while maintaining high frequency resolution in low-frequency areas [24]. This makes the STFT a powerful tool for analysing complex dynamic systems, such as vehicle responses of VBI systems. However, these linear TFR methods are limited by the Heisenberg uncertainty principle to obtain high time and frequency resolutions simultaneously [23]. There are a few studies to extract time-varying characteristics of the VBI system from dynamic responses of the vehicle passage over the bridge. Li et al. [25]) investigated time-varying frequencies of the VBI system from dynamic responses of a two-axle vehicle using multisynchrosqueezing transform. Wang et al. [26] extracted the asymmetry of time-varying frequencies from vehicle responses for detecting damage in bridge support bearings using synchroextracting transform. Zhan et al. [27] identified time-varying frequency of simply supported railway bridge using large-mass vehicle response. Recently, Tan et al. [28] presented a nonlinear time–frequency analysis method to extract IFs of the VBI system using second-order synchrosqueezing transform (SST2). SST2 is a nonlinear time–frequency analysis technique based on the STFT, which redistributes the energy in TFR to concentrate on it at more precise time–frequency locations. This technique not only enhances the clarity of TFR but also allows for the reconstruction of precise local time–frequency features from complex signals. SST2 will be used to extract the time-varying characteristics of the VBI system from dynamic responses of the vehicle passage over the bridge in this study. This study mainly utilises SST2 to extract bridge-damage-related features and conduct systematic parametric studies and experimental investigations.

This paper presents a novel drive-by bridge time-varying feature extraction method for bridge damage detection using SST2. The main contributions of this paper are summarised as follows: (1) A new drive-by bridge time-varying feature extraction method is proposed. The sensors are installed on the vehicle as a mobile sensory system. The bridge dynamic information is captured by the sensory system when the vehicle is passing over the bridge. (2) Time-varying characteristics of the VBI system for bridge damage detection are extracted from dynamic responses of the vehicle passing over the bridge using the proposed method. The influence of the measurement noise and bridge road surface roughness is discussed. (3) The effect of different vehicle parameters on time-varying characteristics of VBI systems, such as the vehicle–bridge mass ratio, the vehicle–bridge frequency ratio, and the vehicle speed is studied through numerical simulations and experimental study. (4) The performance of the proposed method is verified through numerical and experimental studies.

The remainder of the paper is organised as follows: the theory of the proposed method introduced in Section 2, the effect of the vehicle parameters on time-varying characteristics is studied in Section 3, numerical simulations for time-varying features of the VBI with different bridge damage scenarios are conducted in Section 4. The experimental study for verifying the proposed method is conducted in Section 5 and the conclusions are summarised in Section 6.

## 2. Methodology

A new drive-by bridge time-varying feature extraction method is introduced in this section. The fundamental theoretical framework for analysing time-variant dynamic coupling effects in vehicle–bridge systems is introduced first. Then a new signal processing approach utilising second-order synchrosqueezing transform (SST2) is proposed to characterise time-varying properties of vehicle–bridge interaction (VBI) dynamics from dynamic responses of the vehicle passage over the bridge.

### 2.1. Vehicle–Bridge Interaction Systems

#### 2.1.1. VBI Modelling

**Figure 1:**
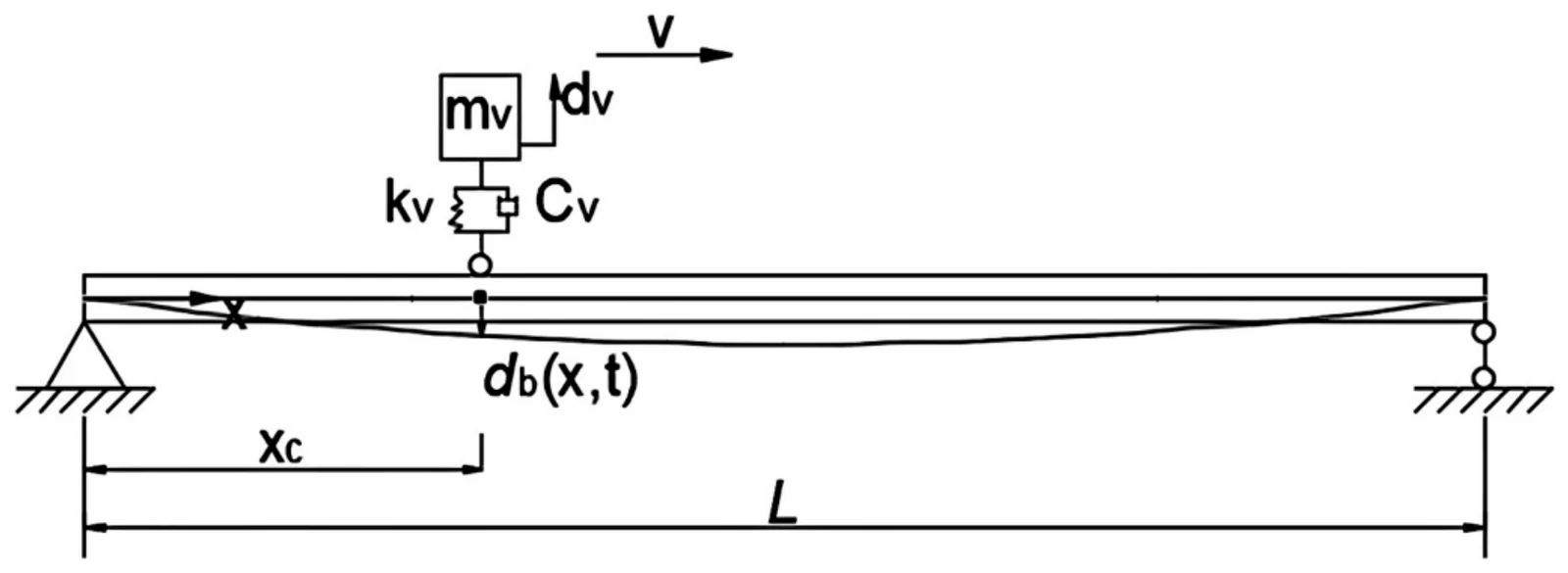
Vehicle–bridge interaction systems.

As shown in Figure 1, the VBI system is represented as a continuous uniform beam bridge subjected to a moving vehicle. The simply supported bridge is modelled using the finite element model. The vehicle is assumed to travel along the longitudinal direction of the bridge at a constant speed v. The vehicle is modelled as a spring mass damper system. The equations of motion for the vehicle subsystem can be expressed as follows:

(1) $$m_{v}{\ddot{d}}_{v}(t)+c_{v}{\dot{d}}_{v}(t)+k_{v}d_{v}(t)=P_{vint}(t)$$

where $m_{v},\;c_{v},\;k_{v}\;$are mass, damping, and stiffness of the vehicle. ${\ddot{d}}_{v}\left(t\right),\;{\dot{d}}_{v}\left(t\right),\;d_{v}(t)$ are vertical acceleration, velocity, and displacement responses of the vehicle respectively. $P_{vint}(t)$ is the interaction force for the vehicle.

The bridge is discretized into finite elements, which is subjected to the time-varying interaction force induced by the moving vehicle. The equations of motion for the bridge subsystem can be expressed as follows:

(2) $$M_{b}{\ddot{d}}_{b}(t)+C_{b}{\dot{d}}_{b}(t)+K_{b}d_{b}(t)=H_{c}(t)(\left.m_{v}g-P_{vint}\left(t\right)\right)$$

where $M_{b},\;C_{b},\;K_{b}\;$are the mass, damping, and stiffness matrices of the bridge respectively. ${\ddot{d}}_{b}\left(t\right),\;{\dot{d}}_{b}\left(t\right),\;d_{b}(t)$ are nodal acceleration, velocity, and displacement vectors of the bridge respectively. $P_{vint}(t)$ is the interaction force from the vehicle. $H_{c}\left(t\right)={\left\{0,0,\ldots ,H_{i}\left(t\right),\ldots ,0\right\}}^{T}$ is a Hermitian cubic interpolation function of time. $H_{i}(t)$ denotes the vector of the shape function in the *i*th element where the moving vehicle is situated at time *t*.

The shape function $H_{i}(t)$ is as follows:

(3) $$H_{i}(t)={\left\{\begin{array}{c} 1-3\xi (t{)}^{2}+2\xi (t{)}^{3},\\ \left(\xi (t)-2\xi (t{)}^{2}+\xi (t{)}^{3}\right)l_{e},\\ 3\xi (t{)}^{2}-2\xi (t{)}^{3},\\ \left(-\xi (t{)}^{2}+\xi (t{)}^{3}\right)l_{e} \end{array}\right\}}^{T}$$

with $\xi (t)-\frac{x(t)-(i-1)l_{e}}{l_{e}}$, where $l_{e}$ is the element length and $(i-1)l_{e}\leq x(t)\leq il_{e}$.

Combining Equations (1) and (2), the equation of motion for the VBI system can be obtained as follows:

(4) $$\;\begin{array}{ll} & \left[\begin{array}{cc} M_{b} & m_{v}H_{c}\\ 0 & m_{v} \end{array}\right]\left\{\begin{array}{c} {\ddot{d}}_{b}\\ {\ddot{d}}_{v} \end{array}\right\}+\left[\begin{array}{cc} C_{b} & 0\\ -c_{v}H_{c}^{T} & c_{v} \end{array}\right]\left\{\begin{array}{c} {\dot{d}}_{b}\\ {\dot{d}}_{v} \end{array}\right\}+\left[\begin{array}{cc} K_{b} & 0\\ -k_{v}H_{c}^{T}-c_{v}{\dot{H}}_{c}^{T} & k_{v} \end{array}\right]\left\{\begin{array}{c} d_{b}\\ d_{v} \end{array}\right\}\\ & =\left\{\begin{array}{c} H_{c}m_{v}g\\ k_{v}r(x)+c_{v}vr^{\prime }(x) \end{array}\right\} \end{array}$$

where $r\left(x\right),\;r^{\prime }\left(x\right)$ are the surface roughness profile of the bridge deck and its first derivative. In Equation (4), the system matrices of the interaction system are time-dependent, determined by the location of the vehicle, and the system’s frequencies vary over time. Dynamic responses of the vehicle across the bridge can be obtained by solving Equation (4) using the Newmark method.

#### 2.1.2. Time-Varying Characteristics of the VBI System

To understand the time-varying characteristics of the VBI system, an approximate analytical solution of instantaneous frequencies for the vehicle and bridge can be obtained when ${\ddot{d}}_{v}\ll g$ as below [7]. When $\omega_{v0}$ > $\omega_{b0}$, instantaneous frequencies of the vehicle and bridge for the VBI system are as follows:

(5) $$\omega_{v}^{2}\left(t\right)=\frac{1}{2}\left(\omega_{v0}^{2}+\omega_{b0}^{2}\right)+\frac{m_{v}\omega_{v0}^{2}}{m_{b}L}\sin^{2}\left(\frac{\pi x_{c}\left(t\right)}{L}\right)+\sqrt{\sqrt{{\left(\omega_{v0}^{2}+\omega_{b0}^{2}+\frac{m_{v}\omega_{v0}^{2}}{m_{b}L}\sin^{2}\left(\frac{\pi x_{c}\left(t\right)}{L}\right)\right)}^{2}-4\omega_{v0}^{2}\omega_{b0}^{2}}}$$

(6) $$\omega_{b}^{2}(t)=\frac{1}{2}\left(\omega_{v0}^{2}+\omega_{b0}^{2}\right)+\frac{m_{v}\omega_{v0}^{2}}{m_{b}L}{sin}^{2}\left(\frac{\pi x_{c}(t)}{L}\right)-\sqrt{\sqrt{{\left(\omega_{v0}^{2}+\omega_{b0}^{2}+\frac{m_{v}\omega_{v0}^{2}}{m_{b}L}\sin^{2}\left(\frac{\pi x_{c}\left(t\right)}{L}\right)\right)}^{2}-4\omega_{v0}^{2}\omega_{b0}^{2}}}$$

When $\omega_{v0}$ < $\omega_{b0}$, instantaneous frequencies of the vehicle and bridge are as follows:

(7) $$\omega_{v}^{2}\left(t\right)=\frac{1}{2}\left(\omega_{v0}^{2}+\omega_{b0}^{2}\right)+\frac{m_{v}\omega_{v0}^{2}}{m_{b}L}\sin^{2}\left(\frac{\pi x_{c}\left(t\right)}{L}\right)-\sqrt{\sqrt{{\left(\omega_{v0}^{2}+\omega_{b0}^{2}+\frac{m_{v}\omega_{v0}^{2}}{m_{b}L}\sin^{2}\left(\frac{\pi x_{c}\left(t\right)}{L}\right)\right)}^{2}-4\omega_{v0}^{2}\omega_{b0}^{2}}}$$

(8) $$\omega_{b}^{2}(t)=\frac{1}{2}\left(\omega_{v0}^{2}+\omega_{b0}^{2}\right)+\frac{m_{v}\omega_{v0}^{2}}{m_{b}L}\sin^{2}\left(\frac{\pi x_{c}\left(t\right)}{L}\right)+\sqrt{\sqrt{{\left(\omega_{v0}^{2}+\omega_{b0}^{2}+\frac{m_{v}\omega_{v0}^{2}}{m_{b}L}{sin}^{2}\left(\frac{\pi x_{c}(t)}{L}\right)\right)}^{2}-4\omega_{v0}^{2}\omega_{b0}^{2}}}$$

where $\omega_{v0}$ is the natural frequency of the vehicle. $\omega_{b0}$ is the natural frequency of the bridge. $m_{v}$ is the mass of the vehicle. $\omega_{v}(t)$ is the instantaneous frequency of the vehicle passing over the bridge. $\omega_{b}(t)$ is the instantaneous frequency of the bridge under the passing vehicle. $m_{b}$ is the mass of the unform bridge. L is the length of the bridge. $x_{c}(t)$ is the position of the vehicle on the bridge as a function of time.

#### 2.1.3. Damage Modelling

In this study, the damage of beam bridges is simulated as a reduction in the beam flexural rigidity as follows [29]:

(9) $$EI(x)=E_{0}I\left(1-\alpha {cos}^{2}\left(\frac{\pi }{2}{\left(\frac{\left|x-l_{c}\right|}{\beta L/2}\right)}^{m}\right)\right),\;\mathrm{for}\;l_{c}-\beta L/2<x<l_{c}+\beta L/2$$

where the damage zone is defined by four parameters, e.g., $\alpha ,\;\beta ,\;l_{c}$, and m. β indicates the relative length of the damage zone along the beam, with possible values between 0.0 and 1.0. α gauges the damage’s intensity, where 0.0 means no damage and 1.0 signifies complete loss of bending stiffness in the middle of the damage zone. The variable $l_{c}$ denotes the midpoint of the damage zone along the beam, starting from the left support. m is related to the damage variation from the centre of the damage zone towards its edges. $E_{0}$ represents the initial modulus of elasticity of the undamaged beam. *I* is the second moment of area.

### 2.2. Time-Varying Feature Extraction Using Second-Order Synchrosqueezing Transform

#### 2.2.1. Synchrosqueezing Transform (SST)

Synchrosqueezing transform (SST) is an extension of traditional time–frequency signal analysis methods like short-time Fourier transform (STFT) or continuous wavelet transform (CWT). The STFT based SST is suitable for signals with closely modulated frequencies, and it provides direct frequency interpretation and a fixed window-based resolution [30]. In this study, the STFT based SST is adopted. Here the response of a vehicle passing over a bridge is expressed as a signal s(t). The multicomponent signal s(t) can be described as follows:

(10) $$s\left(t\right)=\sum_{k=1}^{N}s_{k}(t)=\sum_{k=1}^{N}A_{k}(t)e^{i\phi_{k}(t)}$$

where $s_{k}(t)$ is the kth component of the signal, which is described as the kth intrinsic mode function (IMF). $A_{k}\left(t\right),\phi_{k}(t)$ are the instantaneous amplitude and phase of the kth component respectively. *N* is the number of components in the signal.

By incorporating a window function $g(t)\in L^{2}(R)$ and taking the Fourier transform, the STFT of the signal, denoted as S(t,ω), is computed at a specific time t and frequency ω as follows:

(11) $$S\left(t,\omega \right)=\sum_{k=1}^{N}S_{k}(t,\omega )=\sum_{k=1}^{N}\;\int_{-\infty }^{\infty }\;A_{k}(t+\tau )e^{i\phi_{k}(t+\tau )}g(\tau )e^{-i\omega \tau }d\tau$$

For a slow varying signal, there exists a small enough value ε, $\left|{A\prime }_{k}\left(t\right)\right|\leq \varepsilon ,\left|{\phi ”}_{k}(t)\right|\leq \varepsilon \forall t$. Here, ${A^{\prime }}_{k}(t)$ and ${\phi^{”}}_{k}(t)$ are the first derivative of the amplitude function $A_{k}\left(t\right)$ and the second derivative of the phase function $\phi_{k}(t)$, respectively. Under this assumption, each IMF can be approximate as a purely harmonic signal in a short time. According to the Taylor expansion, the *k*th IMF can be expressed as $s_{k}\left(t+\tau \right)=A_{k}\left(t+\tau \right)e^{i\phi_{k}\left(t+\tau \right)}\approx A_{k}\left(t\right)e^{i\left(\phi_{k}\left(t\right)+{\phi^{\prime }}_{k}\left(t\right)\tau \right)}$. Then Equation (11) can be approximated as follows:

(12) $$S(t,\omega )=\sum_{k=1}^{N}\;A_{k}(t)e^{i\phi_{k}(t)}\int_{-\infty }^{\infty }\;g(\tau )e^{-i\left(\omega -\phi_{k}^{\prime }(t)\right)\tau }d\tau$$

where $\phi_{k}^{\prime }(t)$ is the first derivative of the k-th IMF’s phase, representing the instantaneous frequency of the k-th component at time t. It is estimated as follows:

(13) $${\hat{\omega }}_{k}\left(t,\omega \right)=\phi_{k}^{\prime }(t)=\frac{\partial_{t}S_{k}\left(t,\omega \right)}{iS_{k}(t,\omega )}$$

where $\hat{\omega }(t,\omega )$ is the instantaneous frequency at time t and frequency ω. $\partial_{t}S_{k}\left(t,\omega \right)=\frac{\partial S_{k}(t,\omega )}{\partial t}$.

Here SST is implemented through a frequency reassignment operator to concentrate the dispersed time–frequency coefficients of STFT results to a compact region near the instantaneous frequency trajectories of each mode. The formula is obtained as follows [31]:

(14) $$sst\left(t,\omega \right)=\int_{-\infty }^{\infty }\;S(t,\eta )\delta \left(\omega -\hat{\omega }(t,\eta )\right)d\eta$$

where δ is the Dirac function.

#### 2.2.2. Second-Order Synchrosqueezing Transform (SST2)

As shown in Equation (12), the second derivative of the phase function ${\phi^{”}}_{k}(t)$ could be ignored when the signal is slow-varying. For the signal with high frequency modulation, the second derivative of the phase needs to be considered to capture the higher order variations in the frequency and time of the signal. A second-order local modulation operator for any (t,ω) is defined as follows [31]:

(15) $${\tilde{q}}_{k}\left(t,\omega \right)=\frac{\partial_{t}{\tilde{\omega }}_{k}\left(t,\omega \right)}{\partial_{t}{\tilde{\tau }}_{k}(t,\omega )}=\frac{\partial_{t}\left(\frac{\partial_{t}S_{k}\left(t,\omega \right)}{S_{k}(t,\omega )}\right)}{i-\partial_{t}\left(\frac{\partial_{\omega }S_{k}\left(t,\omega \right)}{S_{k}(t,\omega )}\right)},\;\mathrm{when}\;S_{k}(t,\omega )\neq 0\;\mathrm{and}\;\partial_{t}\left(\frac{\partial_{\omega }S_{k}\left(t,\omega \right)}{S_{k}(t,\omega )}\right)\neq i$$

where ${\tilde{\omega }}_{k}\left(t,\omega \right)$, ${\tilde{\tau }}_{k}\left(t,\omega \right)$ are new complex reassignment operators. ${\tilde{\omega }}_{k}\left(t,\omega \right)=\frac{\partial_{t}S_{k}\left(t,\omega \right)}{iS_{k}(t,\omega )}$, ${\tilde{\tau }}_{k}\left(t,\omega \right)=t-\frac{\partial_{\omega }S_{k}\left(t,\omega \right)}{iS_{k}(t,\omega )}$.

The new second-order IF estimate, ${\tilde{\omega }}_{f}^{\left(2\right)}(t,\omega )$, is defined as follows:

(16) $${\tilde{\omega }}_{k}^{\left(2\right)}\left(t,\omega \right)=\left\{\begin{array}{c} {\tilde{\omega }}_{k}\left(t,\omega \right)+{\tilde{q}}_{k}\left(t,\omega \right)\left(t-{\tilde{\tau }}_{k}\left(t,\omega \right)\right),\;if\;{\partial_{t}\tilde{\tau }}_{k}\left(t,\omega \right)\neq 0\\ {\tilde{\omega }}_{k}\left(t,\omega \right),\;\;\;\;\;\;\;\;\;\;\;\;\;\;\;\;\;\;\;\;\;\;\;\;\;\;\;\;\;\;\;\;\;\;\;\;otherwise \end{array}\right.$$

From Equation (16), the second-order instantaneous frequency ${\hat{\omega }}_{k}^{\left(2\right)}\left(t,\omega \right)$ is obtained as the real part of ${\tilde{\omega }}_{k}^{\left(2\right)}\left(t,\omega \right)$.

Similar to Equation (14), SST2 for any $\left|S\left(t,\eta \right)\right|>\gamma$ is given as follows:

(17) $$sst2\left(t,\omega \right)=\int_{-\infty }^{+\infty }S(t,\eta )\delta \left(\omega -{\hat{\omega }}^{\left(2\right)}(t,\eta )\right)d\eta$$

where the parameter γ serves as a threshold for focusing on energy concentration. SST2 enhances the clarity and precision of the time–frequency representation of signals, especially those with rapidly varying frequencies, making it a valuable tool in areas like audio signal processing, seismic analysis, and vehicle–bridge interaction studies.

#### 2.2.3. Ridge Detection

SST2 has been introduced in Section 2.2.2 and the time–frequency representation (TFR) could be obtained by SST2. Ridge detection is a stable visual feature extraction technique that can extract higher resolution information from TFR [32]. It is normally used to get the optimal frequency curve Ω(t) from TFR, and the energy under this frequency curve is maximised while satisfying a smoothness constraint, enforced through a total variation penalization term. The formula is as follows:

(18) $$\overset{ˆ}{\Omega }=\underset{\Omega }{argmax}\left(\int_{R}\;\;{\left|S(t,\Omega (t))\right|}^{2}dt-\lambda \int_{R}\;\;{\left|\frac{d\Omega (t)}{dt}\right|}^{2}dt\right)$$

In this formula, S(t,Ω(t)) represents the squared magnitude of the TFR at time t and frequency Ω(t). Here, the first term $\int_{R}\;{\left|S(t,\Omega (t))\right|}^{2}dt$ represents the total energy along the frequency curve Ω(t) within the entire signal range R, and the second term $-\lambda \int_{R}\;{\left|\frac{d\Omega (t)}{dt}\right|}^{2}dt$ is the integral of the square of the rate of change in the frequency curve, with λ adjusting the strength of the smoothness constraint. In this study, the ridge detection is used to extract time-varying characteristics of VBI systems.

### 2.3. Validation of SST2

To show the performance of SST2 for time–frequency analysis, the following non-stationary signal is used as an example.

(19) $$x\left(t\right)=\cos\left(2\pi \times \left(0.1t^{2.8}+3\sin\left(2t\right)+10t\right)\right)+e^{-0.2t}cos(2\pi \times \left(60+t^{1.4}\right)t)$$

As listed in Equation (19), there are two time-varying components in the signal. The amplitude and phase of the first time-varying component are $A_{1}\left(t\right)=1,\;\phi_{1}\left(t\right)=0.1t^{2.8}+3\sin\left(2t\right)+10t$ respectively. The amplitude and phase of the second frequency component are $A_{2}\left(t\right)=e^{-0.2t},\;\phi_{2}\left(t\right)=\left(60+t^{1.4}\right)t$. The instantaneous frequencies (IFs) of these two components are ${\phi^{\prime }}_{1}\left(t\right)=0.28t^{1.8}+6\cos\left(2t\right)+10,\;{\phi^{\prime }}_{2}\left(t\right)=60+2.4t^{1.4}$ respectively.

**Figure 2:**
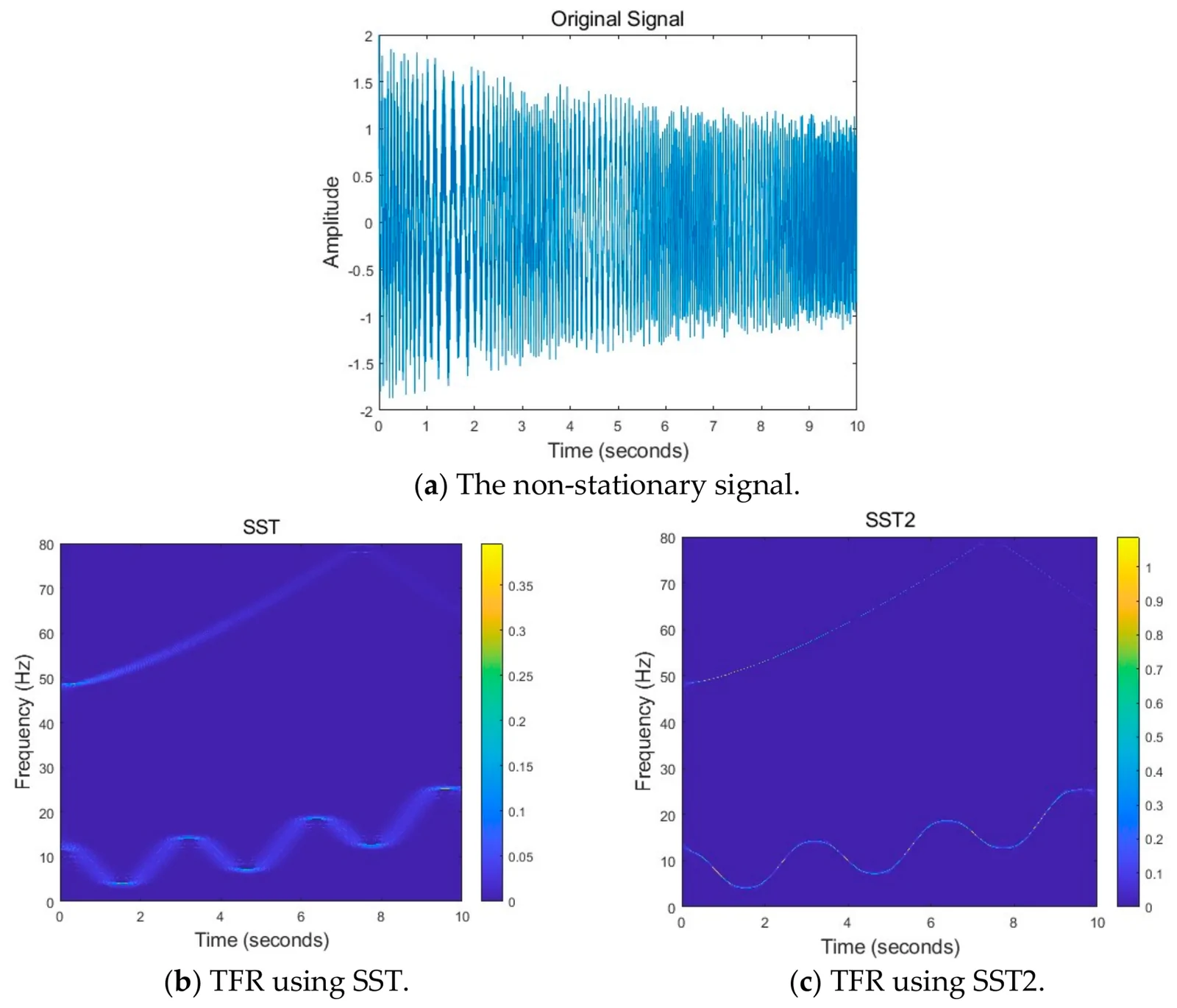
Non-stationary signal under SST and SST2.

Figure 2 shows the non-stationary signal, and its TFRs using SST and SST2. From Figure 2, there are two clear curves corresponding to IFs of those two components in the signal. The results show that both SST and SST2 could extract IFs of the signal. Compared with the results by SST and SST2, the curves in Figure 2c are much thinner than that in Figure 2b. The results show that IFs by SST2 have a high resolution. This is due to SST2 incorporating the second-order derivative information, which significantly enhanced the concentration of its TFR. This improvement contributes to its precise tracking of rapid frequency variations over time, reduces the ambiguity and increases the resolution.

## 3. Numerical Study

### 3.1. Dynamic Responses of VBI Systems

In this section, the bridge is modelled as a simply supported beam with 30 m long. The flexural rigidity and mass per unit length of the bridge are $2.5\times 10^{10}$ Nm^2^ and 6000 kg/m respectively. The first two natural frequencies of the bridge are 3.56 Hz and 14.26 Hz respectively. The bridge is discretized into 10 beam elements. Rayleigh damping is considered with $C_{b}=\alpha_{1}M_{b}+\alpha_{2}K_{b}$ and $\alpha_{1}=0.243,\;\alpha_{1}=0.0001$, where $M_{b},\;C_{b}$ and $K_{b}$ are the mass, damping and stiffness matrices of the bridge respectively. In practice, these two coefficients could be determined by the first two frequencies of the bridge. The quarter-vehicle model with a single degree of freedom is employed. The mass, stiffness and damping of the vehicle are 7000 kg, $2.82\times 10^{6}$ N/m, and 390 Ns/m respectively. The vehicle natural frequency is 3.20 Hz.

In this study, the response of the VBI system is obtained by solving Equation (4) using the Newmark-β method with a time step 0.002 s. The vehicle speed is 2 m/s. The bridge road surface roughness is considered as Class A, which is a smooth condition [33]. To simulate measurements, the white noise is added to the calculated acceleration responses as follows:

(20) $$acc_{m}=acc_{cal}+E_{p}*N_{\mathrm{oise}\;}*\sigma \left(acc_{cal}\right)$$

where $acc_{cal}$ is the calculated acceleration response. $N_{\mathrm{oise}\;}$ is the white noise with a standard normal distribution of a zero mean and a variation in one. $\sigma \left(acc_{cal}\right)$ is the standard deviation of the acceleration response. $E_{p}$ is the noise level.

**Figure 3:**
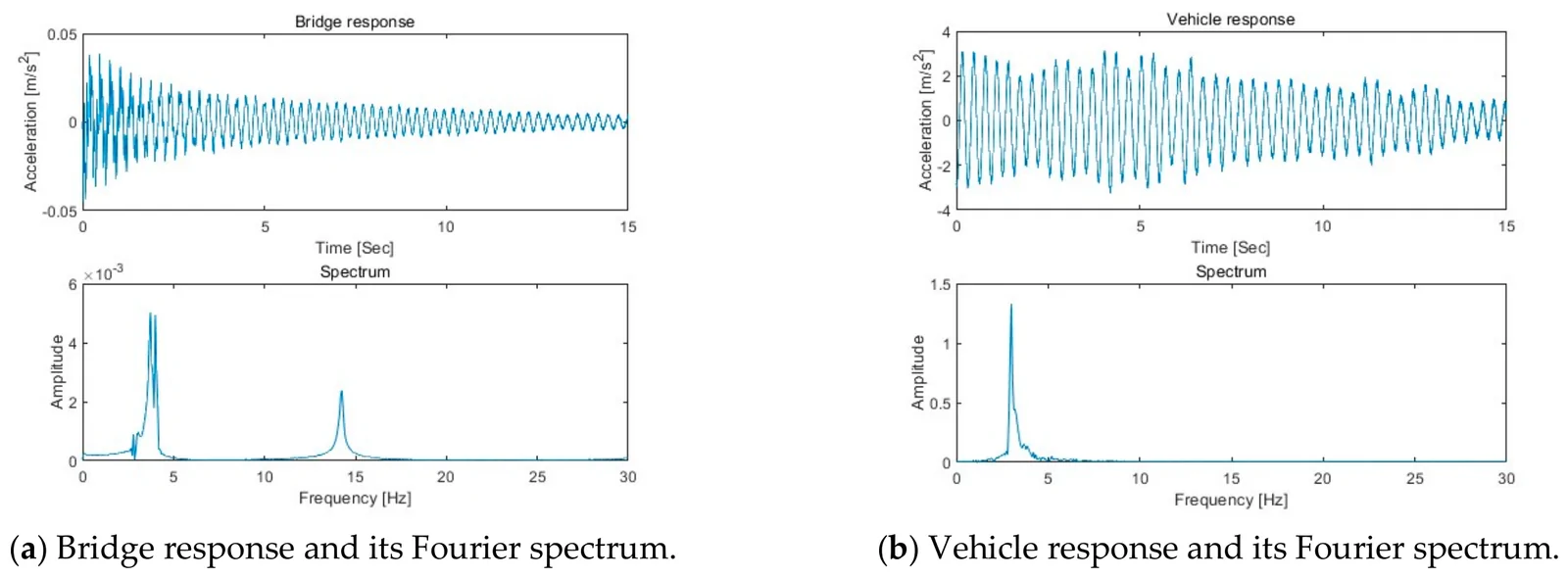
Dynamic responses of VBI systems.

As an example, Figure 3a shows the bridge acceleration response at 3/10L from the left end and its Fourier spectrum. As a general case, 5% noise level has been added in the response. From the Fourier spectrum of the bridge response, two main peaks were found at 3.55 Hz and 13.40 Hz. These two peaks correspond to the first two natural frequencies of the bridge. Figure 3b shows the vehicle response and its spectrum, and there is one peak at 3.20 Hz in the spectrum. The peak corresponds to the natural frequency of the vehicle. From Figure 3b, the vehicle’s own vibration component is dominated, and this overshadows the bridge’s dynamic response components, making it difficult for drive-by inspection even with the smooth road condition.

### 3.2. Time–Frequency Analysis with SST and SST2

In this section, both SST and SST2 have been used to extract the time-varying characteristics of the VBI system from dynamic responses of the passing vehicle, and the results by these two methods are compared. In this section, 5% noise level is added in responses to simulate the measurements.

**Figure 4:**
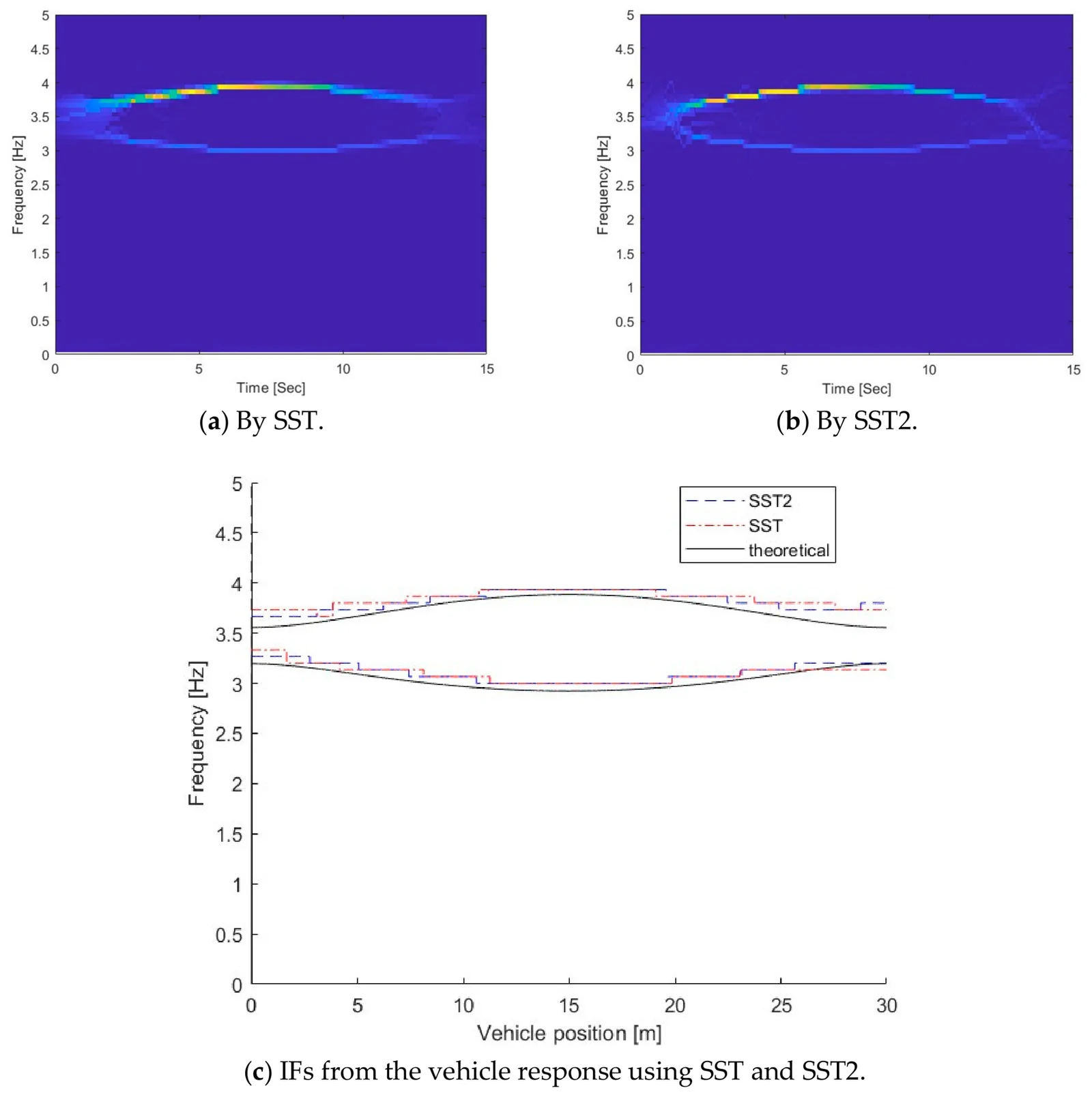
TFRs and IFs from the vehicle response using SST and SST2.

Figure 4 shows TFRs from the vehicle response by SST and SST2, and their corresponding IFs extracted using ridge detection. In Figure 4a,b, there are two trajectories around 3.5 Hz in TFRs. As the vehicle frequency is 3.2 Hz and the first natural frequency of the bridge is 3.56 Hz, the vehicle frequency is smaller than the first natural frequency of the bridge. By Equations (7) and (8), the time-varying frequency above 3.5 Hz is related to the bridge subsystem and the frequency component below 3.5 Hz is related to the vehicle subsystem. From Figure 4a,b, the trajectory below 3.5 Hz is a little bit lighter than that above 3.5 Hz. The results show that the energy of the bridge frequency component is much higher than that of the vehicle frequency component using SST and SST2. Therefore, the time-varying characteristics of the bridge under a moving vehicle are extracted successfully when the road surface condition is smooth.

Figure 4c shows IFs from dynamic responses of the passing vehicle using SST and SST2, that are compared with the analytical solutions using Equations (7) and (8). From the figure, the IFs using both SST and SST2 are close to the analytical solution. The IF by SST is much smoother than that by SST2, especially two ends of the IF. It shows that SST2 could capture the abrupt changes at the entrance and exit of the bridge. This is due to the SST2 could extract the high order components as listed in Equation (16). In the following sections, SST2 is used to extract time-varying characteristics of VBI systems.

**Figure 5:**
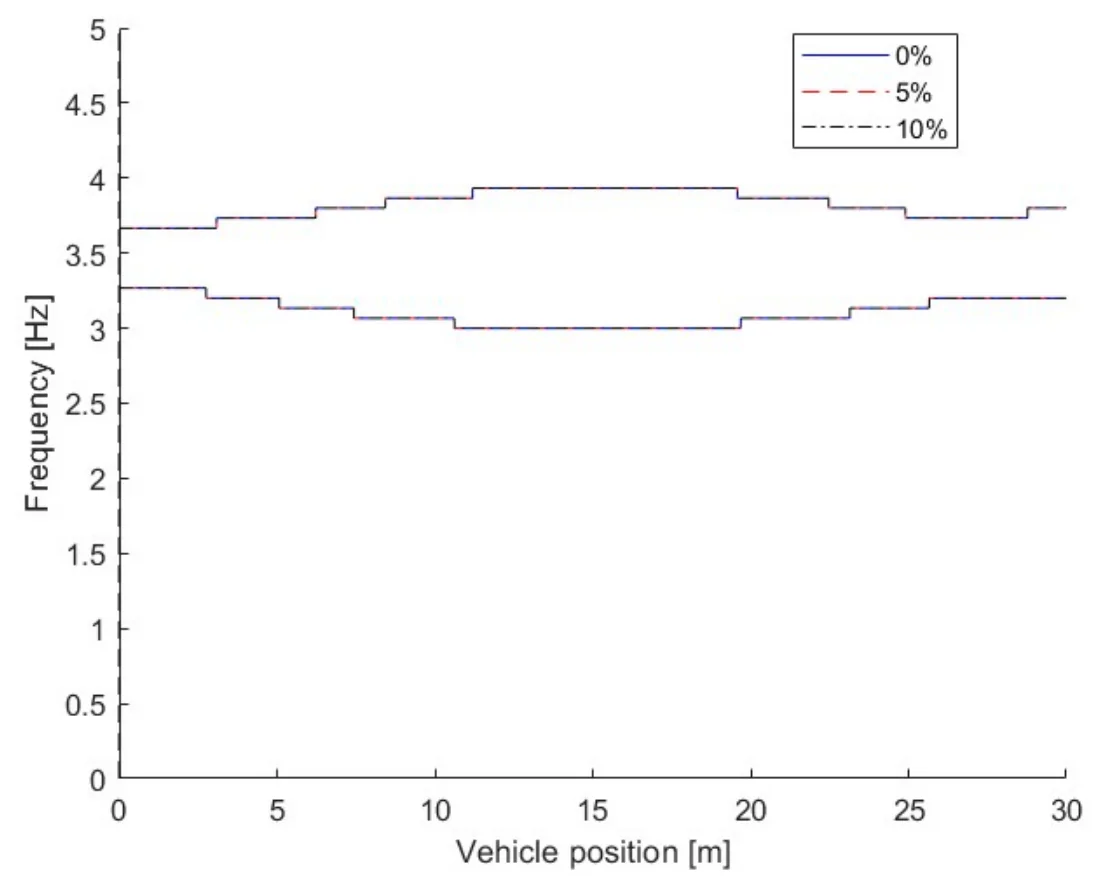
Effect of measurement noise on identified IFs using SST2.

Figure 5 shows the identified IFs from dynamic responses of the passing vehicle with different measurement noise using SST2. A total of 5% and 10% of white noise have been added to the calculated vehicle response respectively. The identified IFs with different measurement noise are compared with that without measurement noise. From the figure, the IFs extracted from the vehicle response with 5% and 10% noise agree well with that without the measurement noise. The results show that the proposed SST2 method is much more robust to measurement noise. As the general case, 5% measurement noise is considered in Section 3.3 and Section 4.

### 3.3. Time-Varying Characteristics of VBI Systems

#### 3.3.1. Effect of Road Surface Roughness

**Figure 6:**
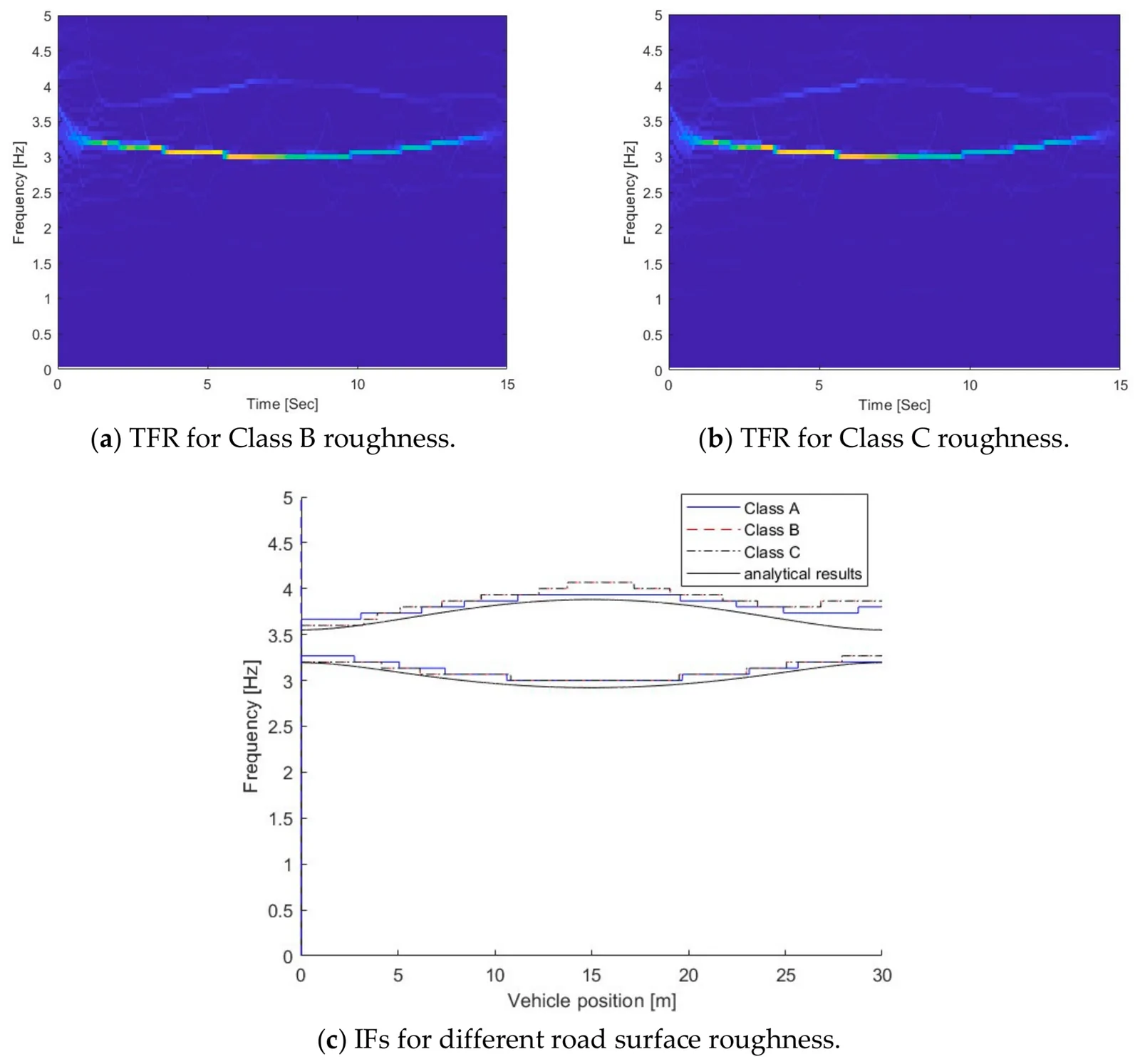
TFRs and their corresponding IFs for different road surface roughness using SST2.

In this section, the effect of road surface roughness on time-varying characteristics of VBI systems is studied using the proposed SST2 method. Three different classes of road surface roughness, e.g., Classes A, B and C are discussed [33]. Other parameters are the same as that in Section 3.1. The results for Class A are shown in Figure 4b. TFRs for Classes B and C road surface roughness are shown in Figure 6a and Figure 6b respectively. Their corresponding IFs are shown in Figure 6c.

From Figure 6a,b, two trajectories around 3.5 Hz could be found. As in Figure 4b, the trajectory above 3.5 Hz corresponds to the bridge response component and that below 3.5 Hz corresponds to the vehicle response component. Compared with the results in Figure 4b, the top trajectories in Figure 6a,b are much lighter than those of the bottom trajectories. The results show that the vehicle response component dominates the dynamic response of the passing vehicle when the road surface roughness is Class B or C.

Figure 6c shows IFs of the VBI system with different road surface roughness. According to the figure, the IFs below 3.5 Hz are related to the vehicle response component and those above 3.5 Hz are related to the bridge response component. The results show that the proposed method is successful to extract both vehicle and bridge IFs from dynamic responses of the vehicle passing over the bridge. The IFs related to the vehicle response component are close each other and it shows that the vehicle IF is not affected by the road surface roughness. The IFs related to the bridge response component are different, and the mean squared error (MSE) values of the difference between the IF and the analytical result for Classes A, B and C are 0.0085, 0.0265 and 0.0265 respectively. In this study, both bridge and vehicle IFs are identified from dynamic responses of the passing vehicle. The results show that the road surface roughness has a slight larger effect on the identified results of bridge IFs than that of vehicle IFs. The bridge IF is the instantaneous frequency of the bridge under the passing vehicle and the vehicle IF is that of the vehicle passing over the bridge. Road surface roughness acts as an external spatial excitation input to the moving vehicle, which heavily excites the natural frequency components of the moving vehicle and often masks weaker bridge response components. The high road roughness amplifies the wideband background energy and alters the bridge instantaneous frequency trajectories in time–frequency representations, which affects the identified results of bridge IFs.

#### 3.3.2. Effect of Vehicle Speeds

**Figure 7:**
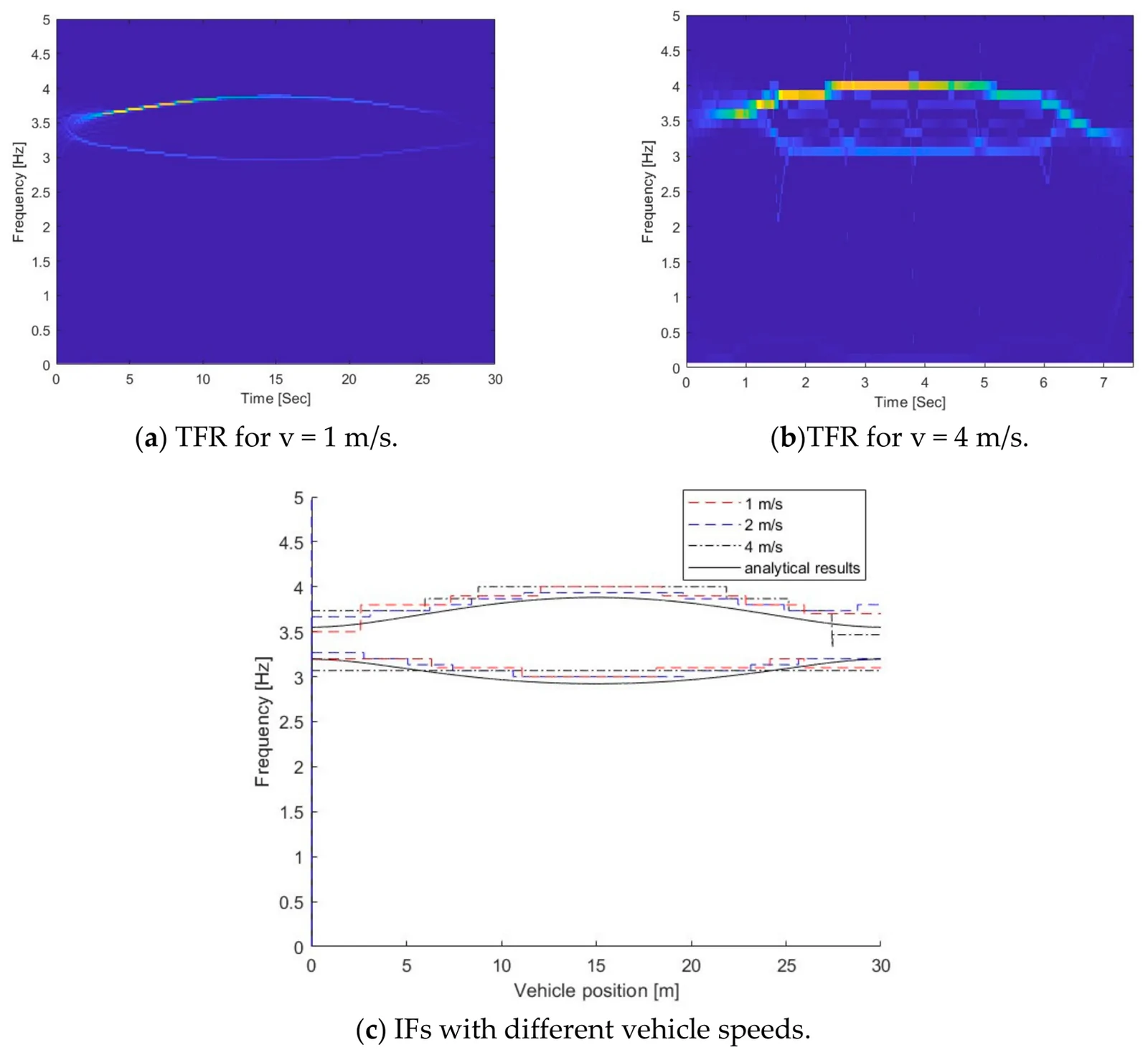
TFRs and IFs with different vehicle speeds.

In this section, time-varying characteristics of VBI systems with different vehicle speeds are studied using SST2. Three different vehicle speeds are discussed, e.g., 1 m/s, 2 m/s and 4 m/s. Other parameters are the same as that in Section 3.1. Figure 7a and Figure 7b show TFRs for vehicle speeds 1 m/s and 4 m/s respectively. The TFR for 2 m/s is shown in Figure 4b. From Figure 7a, Figure 4b and Figure 7b, two trajectories around 3.5 Hz for all speeds are clearly identified, and the top and bottom trajectories correspond to the bridge and vehicle response components respectively. From these figures, the resolution of the TFR reduces when the vehicle speed increases. To get the accurate TFR, it requires a low vehicle speed. Figure 7c shows their corresponding IFs for different vehicle speeds. From the figure, all IFs are close to the analytical solution. When the vehicle speed is 1 m/s, the IF from dynamic responses of the passing vehicle agrees well with the analytical solution. Compared with the analytical solution, the MSE values for 1, 2 and 4 m/s are 0.0024, 0.0085 and 0.0129. The results show that the MSE value increases with the vehicle speed and this is due to the resolution of the IF reduces with the vehicle speed increases.

#### 3.3.3. Effect of Vehicle–Bridge Mass Ratio and Vehicle–Bridge Frequency Ratio

In this section, the effect of the vehicle–bridge mass ratio and the vehicle–bridge frequency ratio on time-varying characteristics of VBI systems is studied. Class A road surface roughness is considered, and the vehicle speed is 2 m/s. 5% noise is added in the calculated response. As listed in Table 1, different vehicle model parameters are studied, e.g., Cases 1–7. Other parameters are the same as that in Section 3.1.

**Figure 8:**
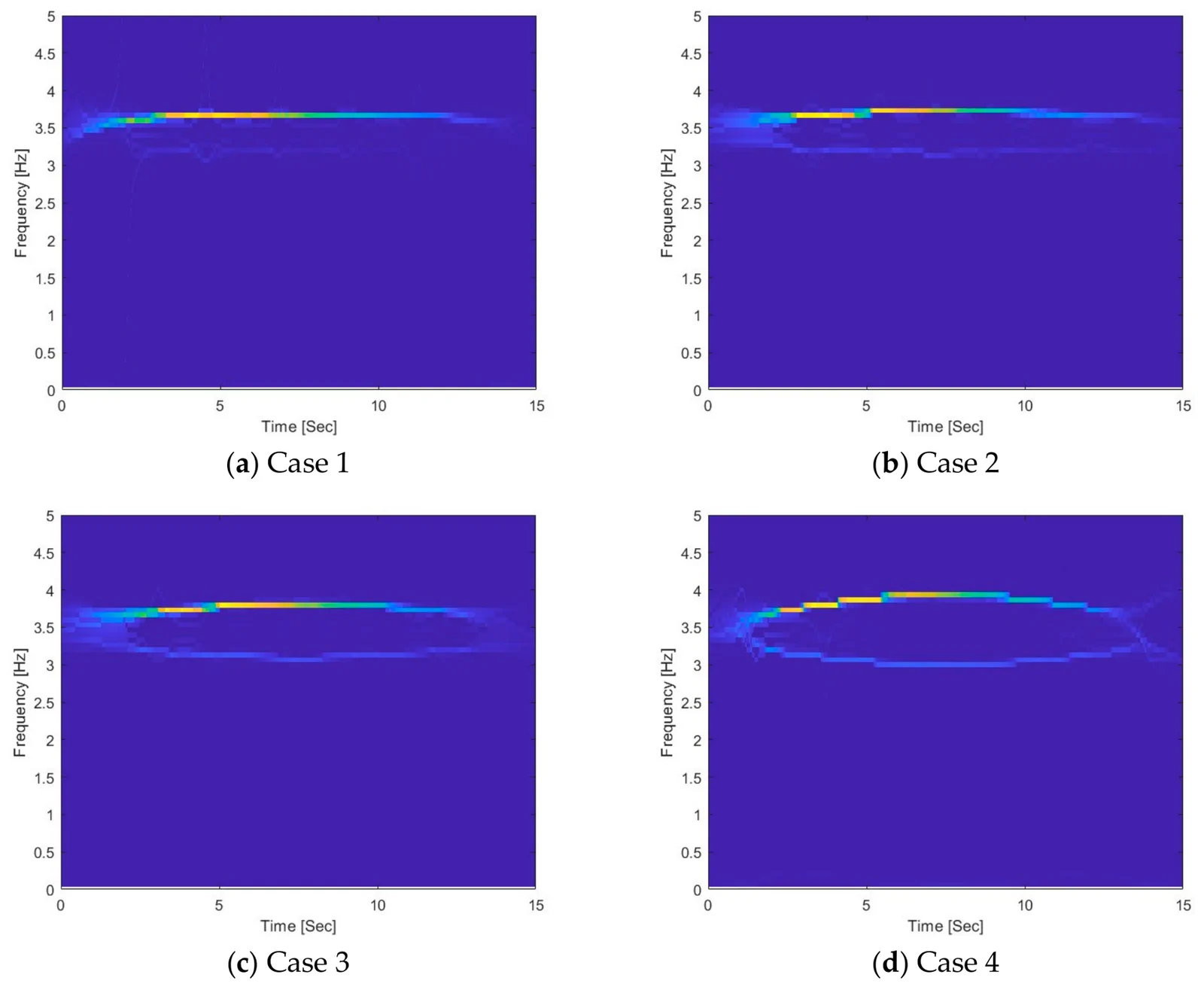
TFRs with different vehicle–bridge mass ratios.

Figure 8 shows TFRs for Cases 1–4 respectively. In these cases, the vehicle–bridge frequency ratios are the same as 0.90, and the vehicle–bridge mass ratios are 0.0048, 0.0095, 0.0190 and 0.390 respectively. From Figure 8, there are two trajectories around 3.5 Hz for all cases. For these cases, the vehicle frequencies are smaller than the bridge frequency. According to Equations (7) and (8), the top trajectory is related to the bridge response component, and the bottom one corresponds to the vehicle response component. From Cases 1 to 4, the bottom trajectory becomes clear while the top trajectory is highlighted. This is due to the energy of the vehicle response component and the vehicle–bridge coupling effect increase with the vehicle–bridge mass ratio, and the bridge response component is dominant in the response of the passing vehicle. A heavy vehicle could enhance the vehicle–bridge coupling effect.

**Figure 9:**
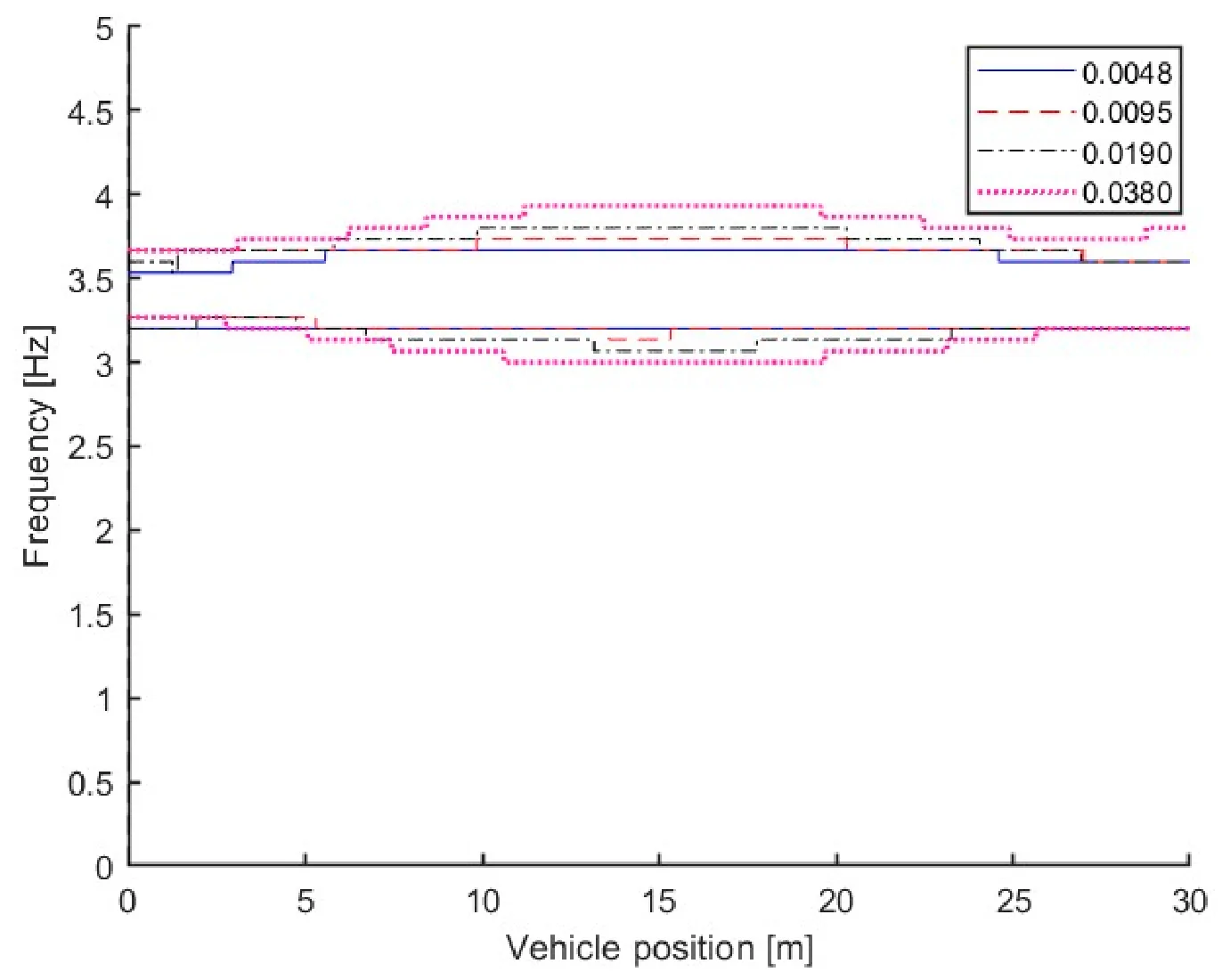
IFs with different vehicle–bridge mass ratios.

Figure 9 shows IFs for Cases 1–4. From the figure, the IFs related to vehicle and bridge response components are extracted clearly. The frequency variation in IFs is increased with the vehicle–bridge mass ratio. The results show that the vehicle–bridge coupling effect is increased when the vehicle–bridge mass ratio increases.

**Figure 10:**
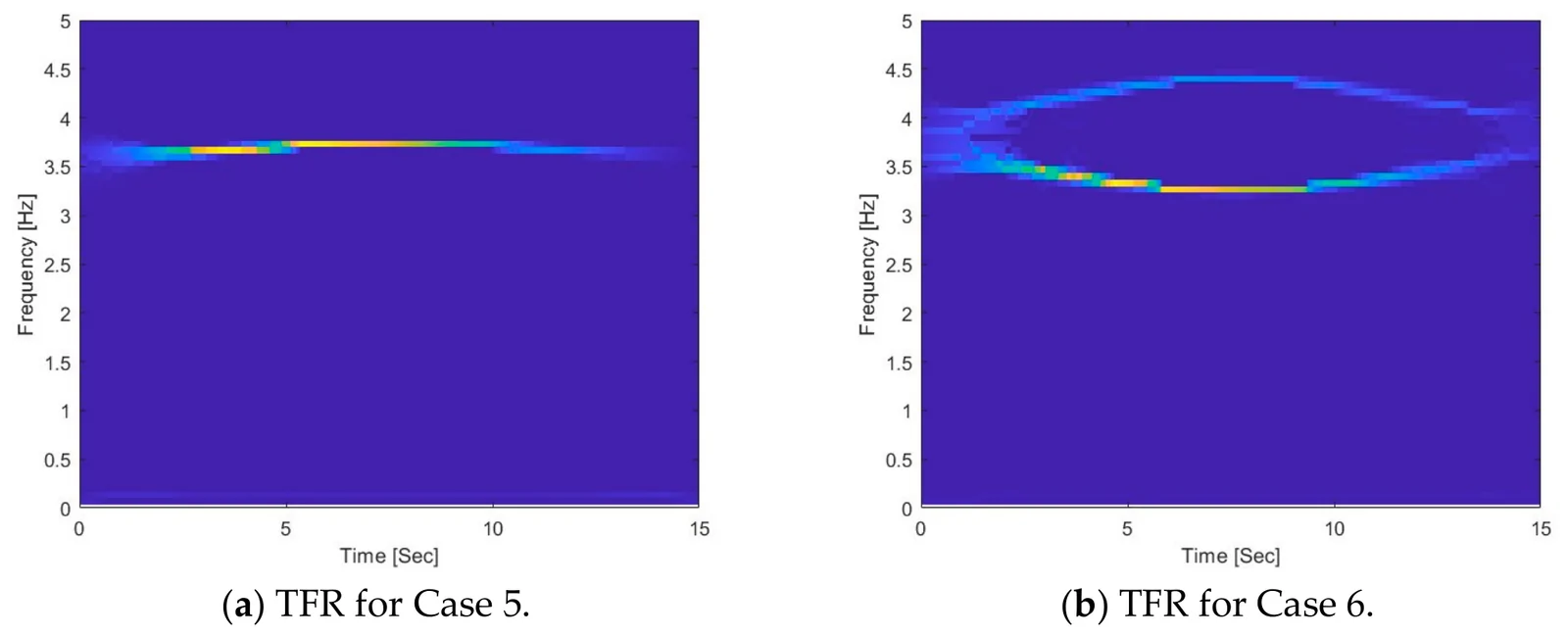

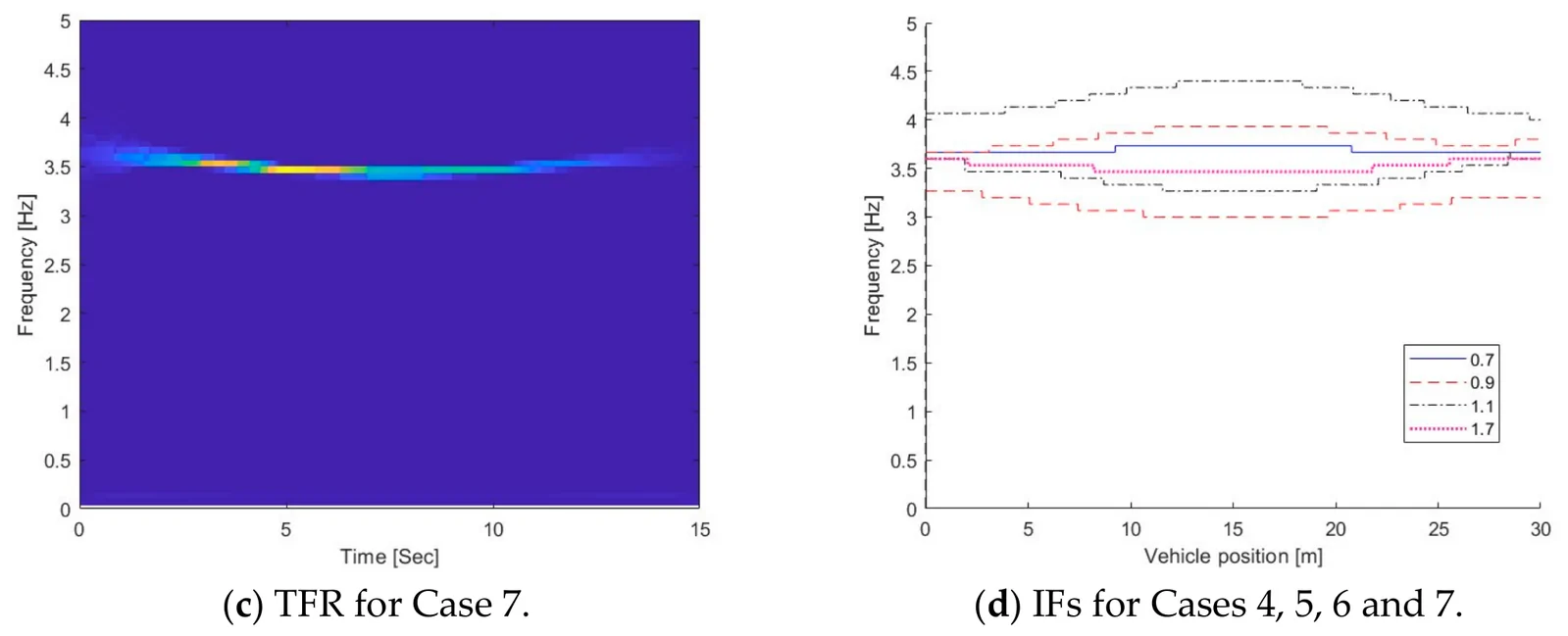
TFRs and IFs with different vehicle–bridge frequency ratios.

For Cases 4–7, the vehicle–bridge mass ratios are the same as 0.0390, and the vehicle–bridge frequency ratios are 0.90, 0.70, 1.10 and 1.70 respectively. TFRs for Cases 4, 5, 6 and 7 are shown in Figure 8d, and Figure 10a, Figure 10b and Figure 10c respectively. Figure 10d shows IFs for Cases 4–7. There are two clear trajectories around 3.5 Hz for Cases 4 and 6 in Figure 8d and Figure 10b. There is only one trajectory above 3.5 Hz for Case 5 in Figure 10a, and only one trajectory below 3.5 Hz for Case 7 in Figure 10c. This is due to the vehicle–bridge coupling effect when the vehicle–bridge frequency ratios for Cases 4 and 6 are 0.90 and 1.10 respectively, close to 1.00. The vehicle–bridge frequency ratios are 0.70 and 1.70 for Cases 5 and 7 respectively, which are far away from 1.00. IFs in Figure 10c further confirm the vehicle–bridge coupling effect, where there are large variations for Cases 4 and 6, and close to 3.5 Hz for Cases 5 and 7.

## 4. Time-Varying Characteristics of VBI Systems for Bridge Damage Detection

Section 3.3 has studied the effect of vehicle parameters on time-varying characteristics. In this section, the effect of bridge damage on time-varying characteristics will be studied for structural damage detection. The vehicle and bridge parameters in Section 3.1 are used in this section. A total of 5% measurement noise is added in the vehicle responses to simulate the measurements. The parameter *m* characterises the variation in the beam flexural rigidity. In this study, the concrete bridge is considered, and a flat damage pattern is simulated, e.g., *m* > 1. For an arbitrary case, *m* = 2 is adopted in this study. Based on Equation (9), the effect of three parameters is studied, e.g., the damage severity parameter α, the damage region parameter *β*, and the damage location $l_{c}$.

### 4.1. Effect of Damage Severity Parameter α

In this section, three different damage severities are studied. Based on Equation (9), the damage severity parameter α is set to 0, 0.3, and 0.5 respectively. Here β=0.1, m=2, and the damage location is one-third span of the bridge, e.g., $l_{c}=L/3$. The dynamic responses of the vehicle cross the bridge are simulated using Equation (4) and the proposed SST2 is used to extract the time-varying characteristics of the VBI system from the vehicle responses.

**Figure 11:**
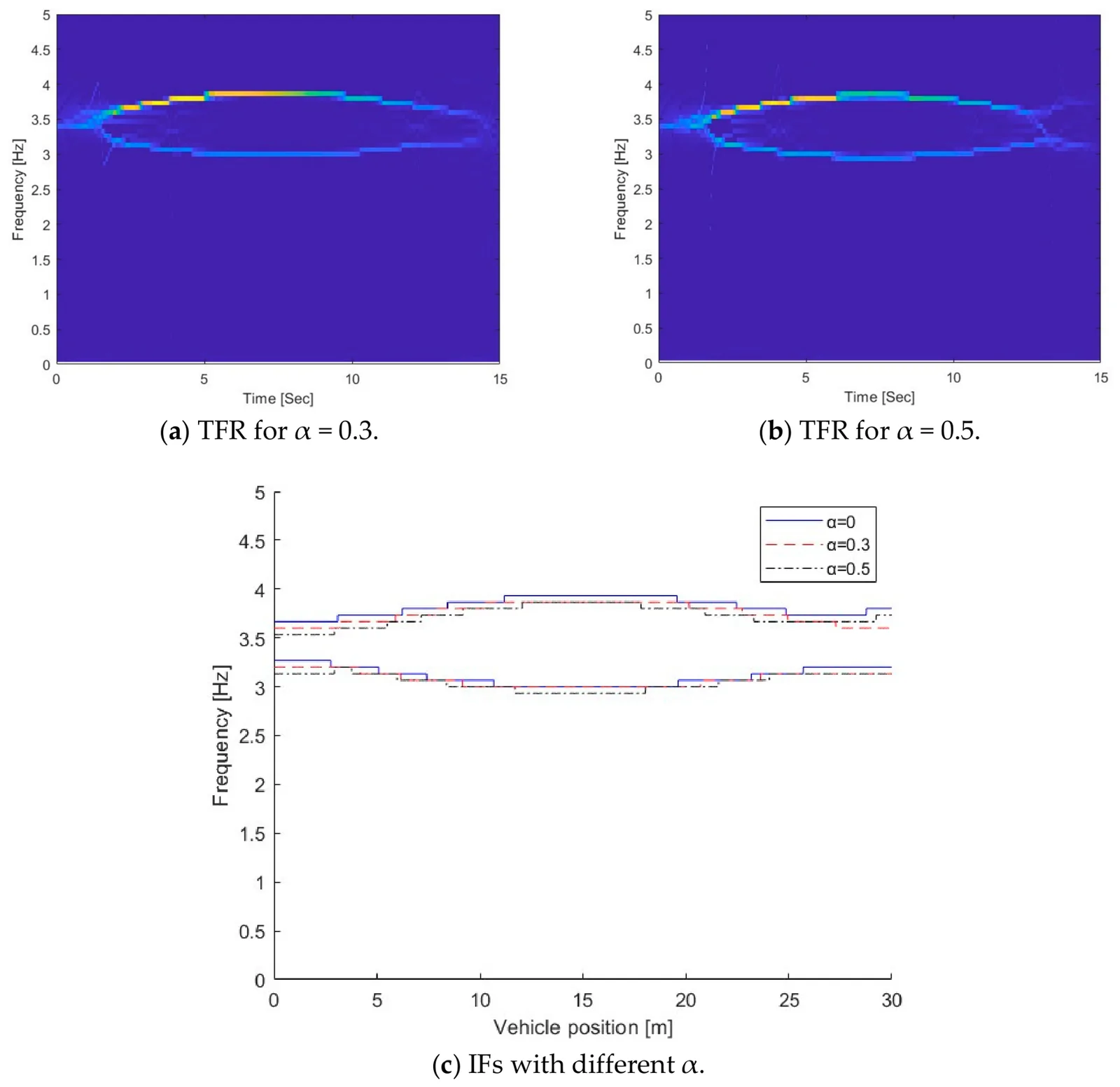
TFRs and IFs for different damage using SST2.

Figure 11a shows the TFR of the vehicle response for the VBI system with small bridge damage scenario (α = 0.3) at the one-third span of the bridge $l_{c}=L/3$, and Figure 11b shows TFR with the large damage scenario (α = 0.5). The results are compared with those without the bridge damage (α = 0), which are shown in Figure 4b. Figure 11c shows IFs of these three scenarios. From TFRs in Figure 4b and Figure 11a,b, there are two clear trajectories around 3.5 Hz. The trajectory above 3.5 Hz corresponds to the bridge response component and that below 3.5 Hz corresponds to the vehicle response component. From the results, the top trajectory becomes light, and the bottom trajectory becomes clear when the bridge damage increases. This is due to the fact that bridge frequency reduces with damage increases. As the vehicle–bridge frequency ratio approaches 1.00, the energy of the vehicle frequency component increases with the vehicle–bridge coupling effect.

Figure 11c shows three IFs corresponding to different bridge damage scenarios using SST2. The dashed blue line represents the instantaneous frequency of the VBI system without damage in the bridge, the dashed red line signifies that with small bridge damage, and the solid blue line indicates that with large bridge damage. From Figure 11c, the trajectories are moving downward with the damage increases as the bridge frequency reduces. From the results, the variation in IF is large when the vehicle is trespassing over the damage location, and the change in IF increases with the damage severity. This is due to the large damage corresponding to a large local stiffness loss around the damage zone, and it induces a large frequency change when the vehicle is trespassing over the damage location.

According to the information presented above, the bridge damage is a local phenomenon, and the instrumented vehicle passing over the damage zone induces the local vibration. The local response is also captured by the passing vehicle. The IFs extracted from the vehicle responses using the proposed SST2 method could be good indicator of the bridge damage.

### 4.2. Effect of Damage Region Parameter β

As listed in Equation (9), the parameter *β* represents the relative length of the damage zone along the beam bridge with a range between 0.0 and 1.0. A small *β* value indicates that the damage is concentrated in a small section of the beam bridge, while a large *β* value suggests a more extensive damage area. To study the effect of the damage range *β*, three damage scenarios are simulated, e.g., 0, 0.1 and 0.25 respectively. Other parameters are constant, e.g., α = 0.3, m = 2 and $l_{c}=L/3$.

**Figure 12:**
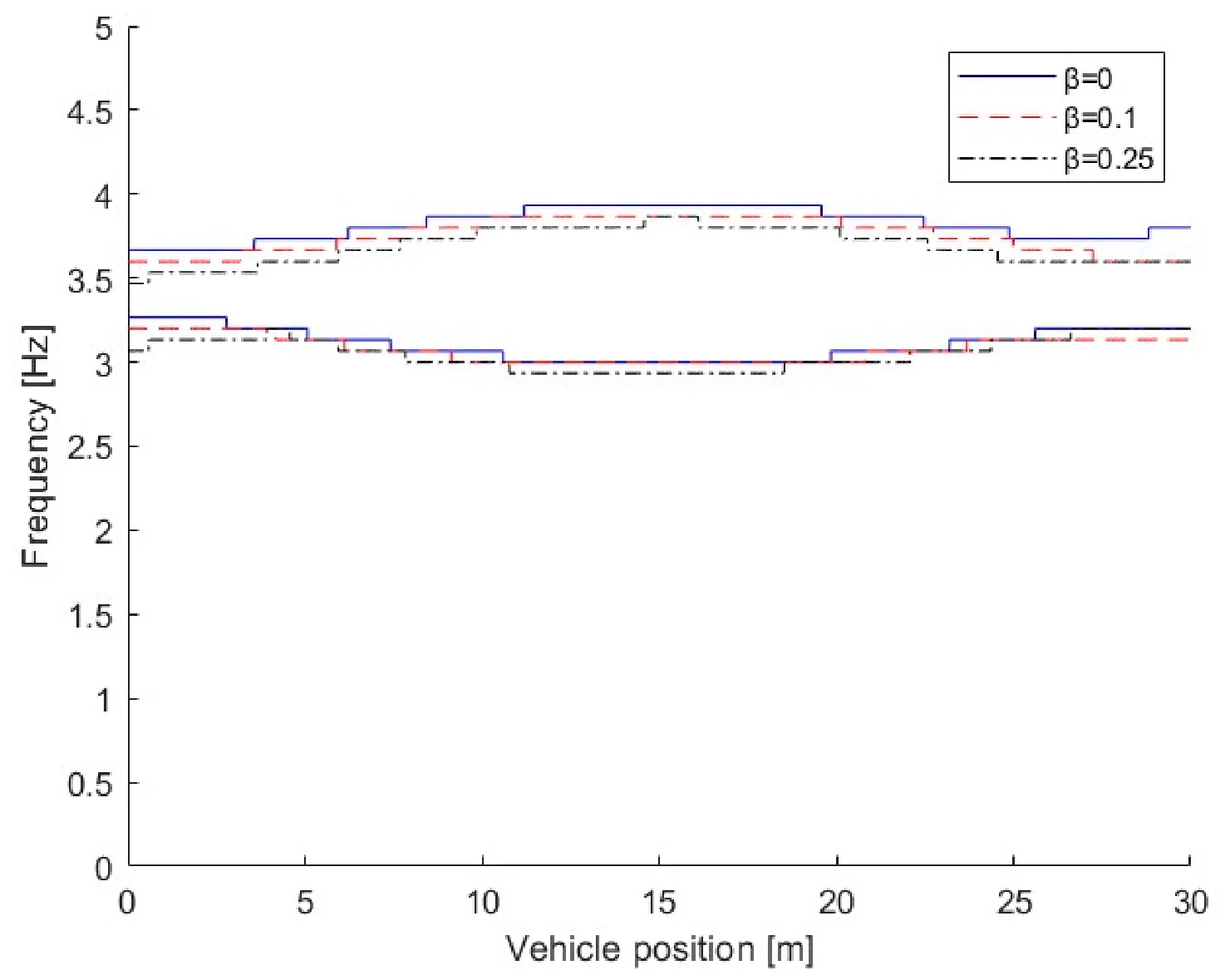
IFs for different *β* using SST2.

Figure 12 shows the IFs for three different damage ranges. From the figure, the overall frequency reduces with the damage range increases. As the bridge damage range increases, the local stiffness around the damage zone is reduced. When the vehicle is passing over the damage location, the IF is reduced, and the change in IF is a good indicator of the bridge damage.

### 4.3. Effect of the Damage Location

**Figure 13:**
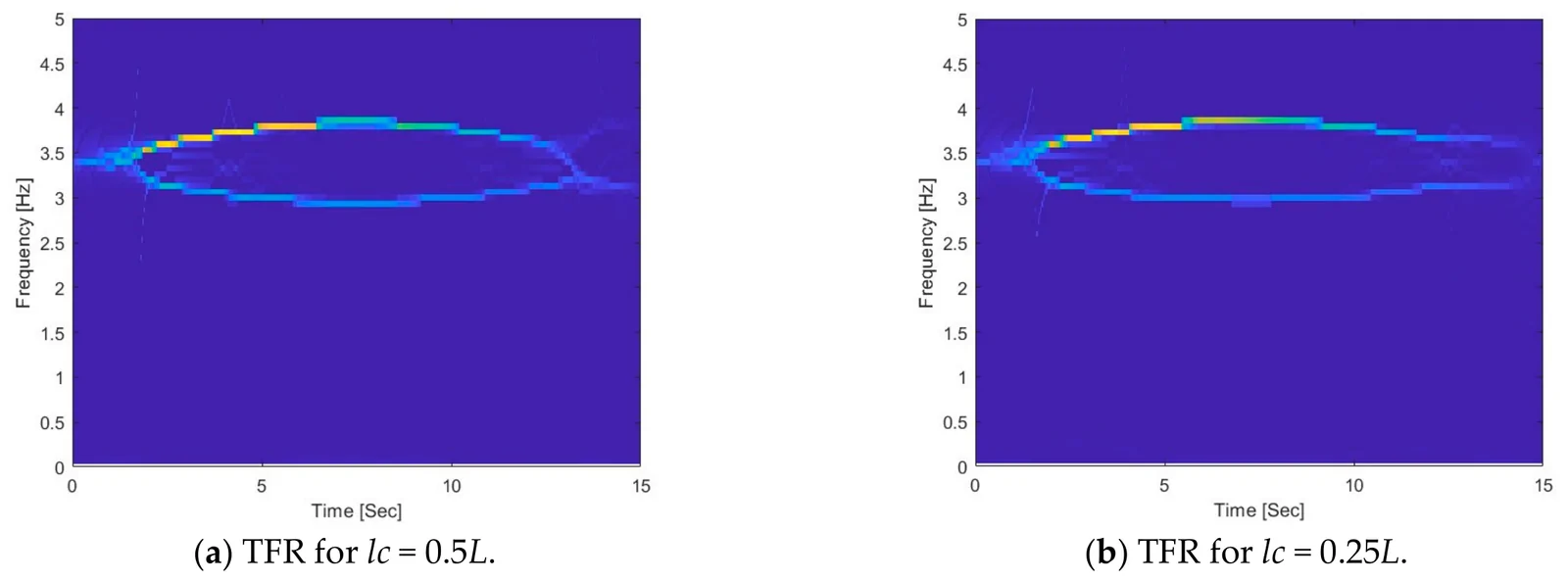

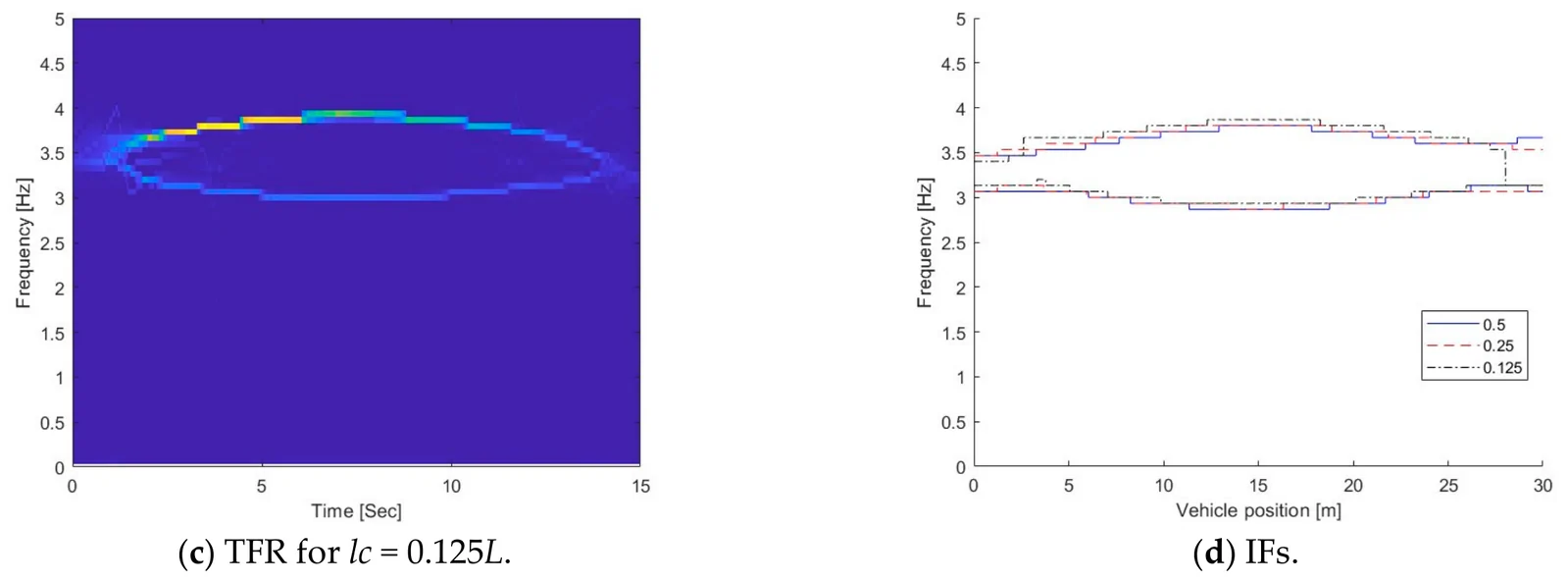
TFRs and IFs for different damage locations.

This section is focused on studying the effect of the bridge damage location. Three different damage scenarios are simulated, e.g., $l_{c}=0.125L,\;0.25L,\;0.5L$ respectively. Here β=0.1, α=0.3, m=2. Other parameters of the VBI system are the same as that in Section 3.1. Figure 13a–c show TFRs for different damage locations using the proposed method. Figure 13d shows their corresponding IFs.

According to Figure 13, there are two clear trajectories around 3.5 Hz in TFRs from dynamic responses of the vehicle passing over the bridge. The first natural frequencies of the bridge with the damage at 1/8 L, 1/4 L and 1/2 L are 3.53 Hz, 3.44 Hz and 3.41 Hz respectively. From Figure 13a–c, the difference between the top and bottom trajectories becomes large around the damage location. The results show that the frequency change is large when the vehicle is around the damage location and this is due to the local response induced by the damage. Figure 13d shows IFs for different damage locations. According to the figure, the bridge damage not only affects the absolute value of IFs but also influences the pattern of the frequency change over the vehicle position. The proposed SST2 method is used to extract the time-varying characteristics from dynamic responses of the vehicle passage over the bridge. For the damage scenario at the mid-span of the bridge, the IF is reduced when the vehicle is around the mid-span of the bridge. For different damage location scenarios, the IFs have large variations when the vehicle is passing over the damage location. According to the results, when the vehicle trespassing a damage section, it creates a localised disruption in the extracted IF profile, mapping the damage location along the span. This is due to the vehicle passing over the damaged location inducing a local response. The local response embeds the effect of the local damage, which is captured by the passing instrumented vehicle. The variation in IFs could be a good indicator of the damage location. In summary, the location of the damage directly and significantly affects the time-varying characteristics of the VBI system, as reflected in changes in IFs. By analysing these time-varying features, we can more accurately assess the health status and structural integrity of the bridge.

### 4.4. Discussion

To explore the quantitative results for different parameters, the mean square errors (MSEs) have been used to quantify the change in IFs related to the reference. The bridge IFs are used in calculation in this study as they directly relate to structural change. Table 2 shows the MSE values for different road roughness, vehicle speeds and measurement noise. There are nine scenarios in total. Scenarios 1, 2 and 3 are corresponding to Classes A, B, C road roughness and Class A, e.g., smooth road roughness is used as the reference. Other parameters of VBI systems are listed in Section 3.3.1. Scenarios 4, 5, and 6 correspond to different vehicle speeds 1 m/s, 2 m/s and 4 m/s respectively, and the parameters are the same as that in Section 3.3.2. Scenario 4 with 1 m/s is used as the reference. Scenarios 7, 8 and 9 are for different measurement noise levels in Section 3.2, and the reference is without measurement noise. All references are noted as “-” in Table 2. From Table 2, the MSE values for Classes B and C road roughness are the same as 0.0028, and the MSE values for 2 m/s and 4 m/s are 0.0036 and 0.0129 respectively. The results show that the road roughness has a light effect on the bridge IF. The bridge IF increases with the vehicle speed and the measurement noise does not have obvious effects on the bridge IF.

Table 3 shows the MSE values for different damage scenarios. Scenarios 10, 11, and 12 are related to different damage severities, e.g., 0.0, 0.3 and 0.5, and other parameters are the same as that in Section 4.1. Scenarios 13, 14 and 16 are related to different damage region parameters in Section 4.3. Scenarios 17, 18 and 19 are related to different damage locations in Section 4.3. Table 3 shows MSE values for different damage scenarios and ‘-’ is noted as the reference. According to Table 3, the MSE value is increased with the damage severity and the damage region parameter. When the damage location is close to support, the MSE value becomes large.

As highlighted above, the bridge IF is much robust to the measurement noise. It is affected slightly by the road roughness, and the vehicle speed has a large effect on the bridge IF. The MSE value increases with the damage severity and damage region parameter, and the damage location also affects the MSE value. Numerical results show that bridge IFs are much sensitive to the bridge damage, and the MSE value could be an indicator of the bridge damage. Further study is needed to determine the criteria for quantifying structural damage.

## 5. Experimental Study

### 5.1. Experimental Setup

**Figure 14:**
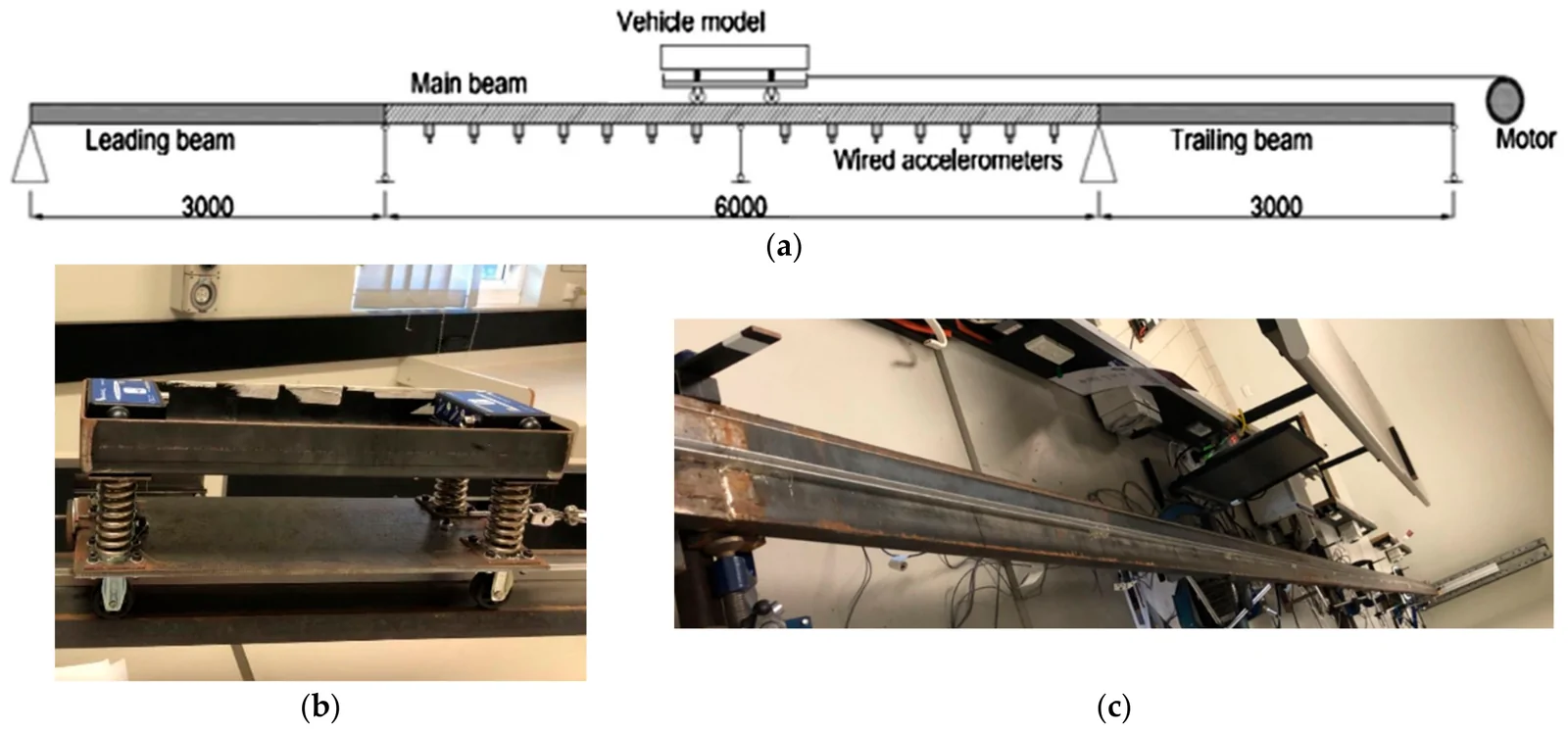
Experimental setup. (**a**) Vehicle–bridge interaction model; (**b**) the vehicle model with wireless accelerometers; (**c**) the bridge model.

To further verify the proposed method, a vehicle–bridge interaction system has been constructed in the laboratory, as depicted in Figure 14a. The bridge model comprises three rectangular steel beams, with the central beam serving as the main beam, measuring 6 m in length and having a cross-section of 100 mm × 15 mm. This main beam is a simply supported structure with two equal spans. Additionally, a leading beam and a trailing beam, each 3 m in length, are positioned in front of and behind the main beam to facilitate the vehicle’s acceleration and deceleration. The first five natural frequencies of the main beam are 5.68 Hz, 8.48 Hz, 13.75 Hz, 18.28 Hz and 24.48 Hz, respectively.

A two-axle vehicle model is used, as shown in Figure 14b. The spacing of two axles is 0.30 m. The mass of the vehicle body is 4.9 kg. The wheels of this vehicle model are made of plastic rubber with a light mass and rigid stiffness. The first natural frequency of the vehicle model is 29.36 Hz with regard to the vehicle bouncing. The vehicle–bridge mass ratio is about 0.069. An electric motor is used to pull the vehicle moving along the test beam bridge. A U-section is installed on the top of the main beam to guide the vehicle moving along the central line of the main beam.

**Figure 15:**
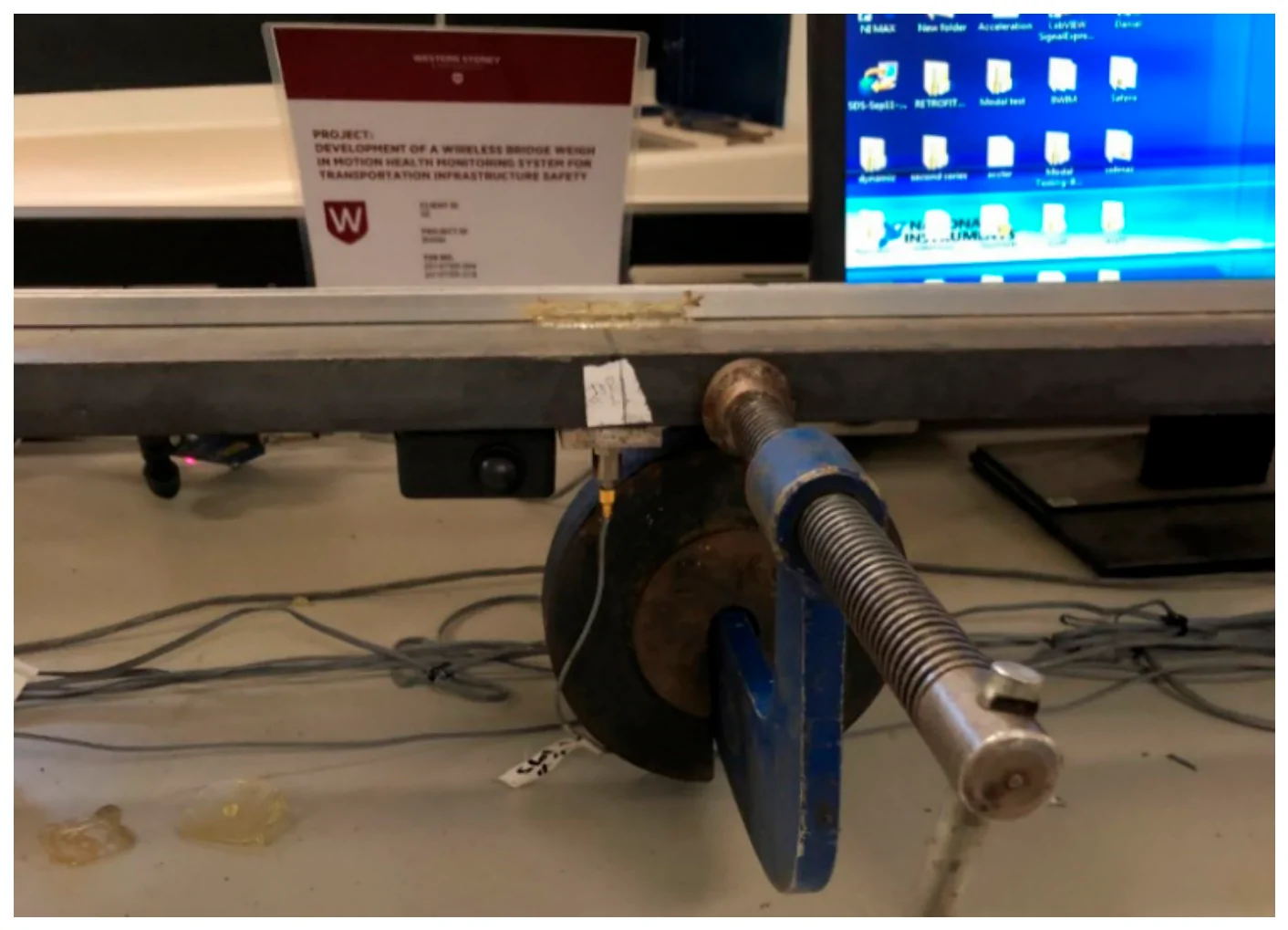
Adding 5 kg mass to the beam.

In this study, an additional 5 kg mass is added at the midpoint of the first span of the main beam to simulate the structural change, as shown in Figure 15. Although the mass change is physically different with the stiffness reduction, both added mass and stiffness reduction induce the change in natural frequencies. This experimental study is dedicated mainly to verifying the sensitivity of extracted instantaneous frequencies to a local structural change rather than the stiffness reduction assumed numerically. A wireless sensing system manufactured by BeanAir, Berlin, Germany is used to monitor dynamic response of the VBI system. Two wireless accelerometers are installed on the vehicle model corresponding to the locations of the front and rear axles respectively, as shown in Figure 14b. Laser sensors are used to record the time when the vehicle enters and exits the main beam, which are then used to estimate the vehicle’s speed. The sampling frequency for the wireless measurement system is 500 Hz.

### 5.2. Experimental Results with Different Vehicle–Bridge Mass Ratios

**Figure 16:**
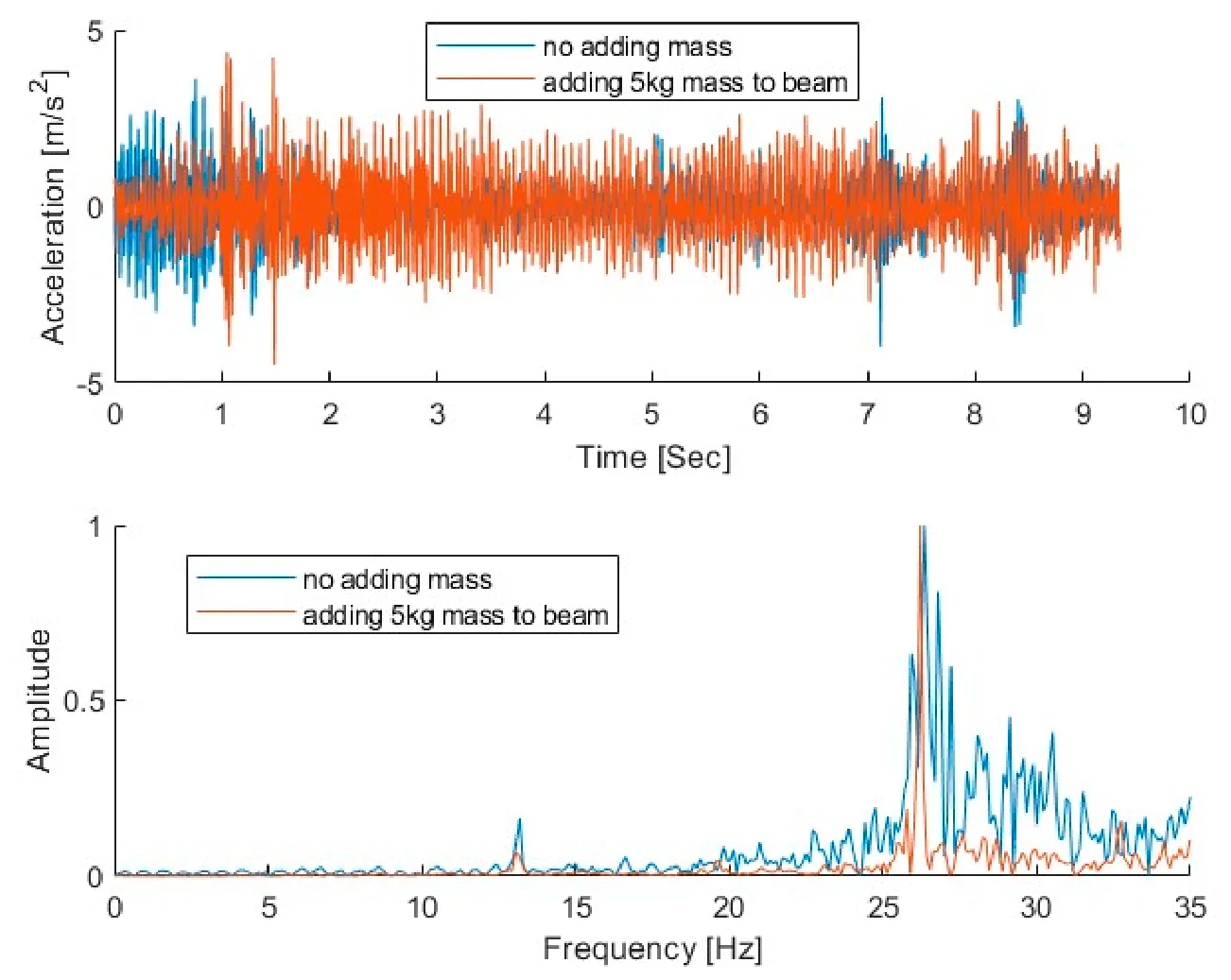
Dynamic responses and their Fourier spectra of the vehicle without 4 kg weight block.

**Figure 17:**
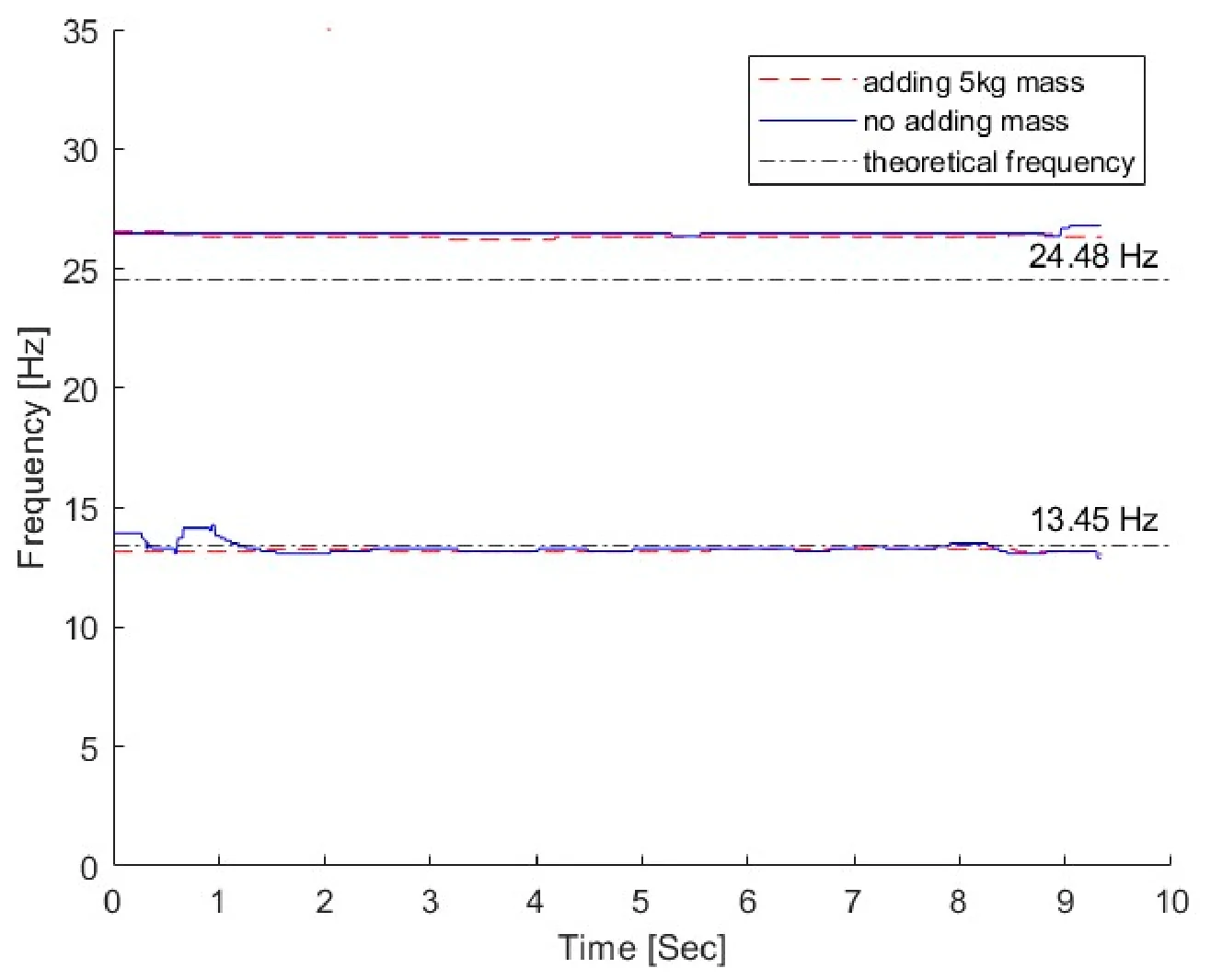
IFs of the vehicle response without 4 kg weight block.

**Figure 18:**
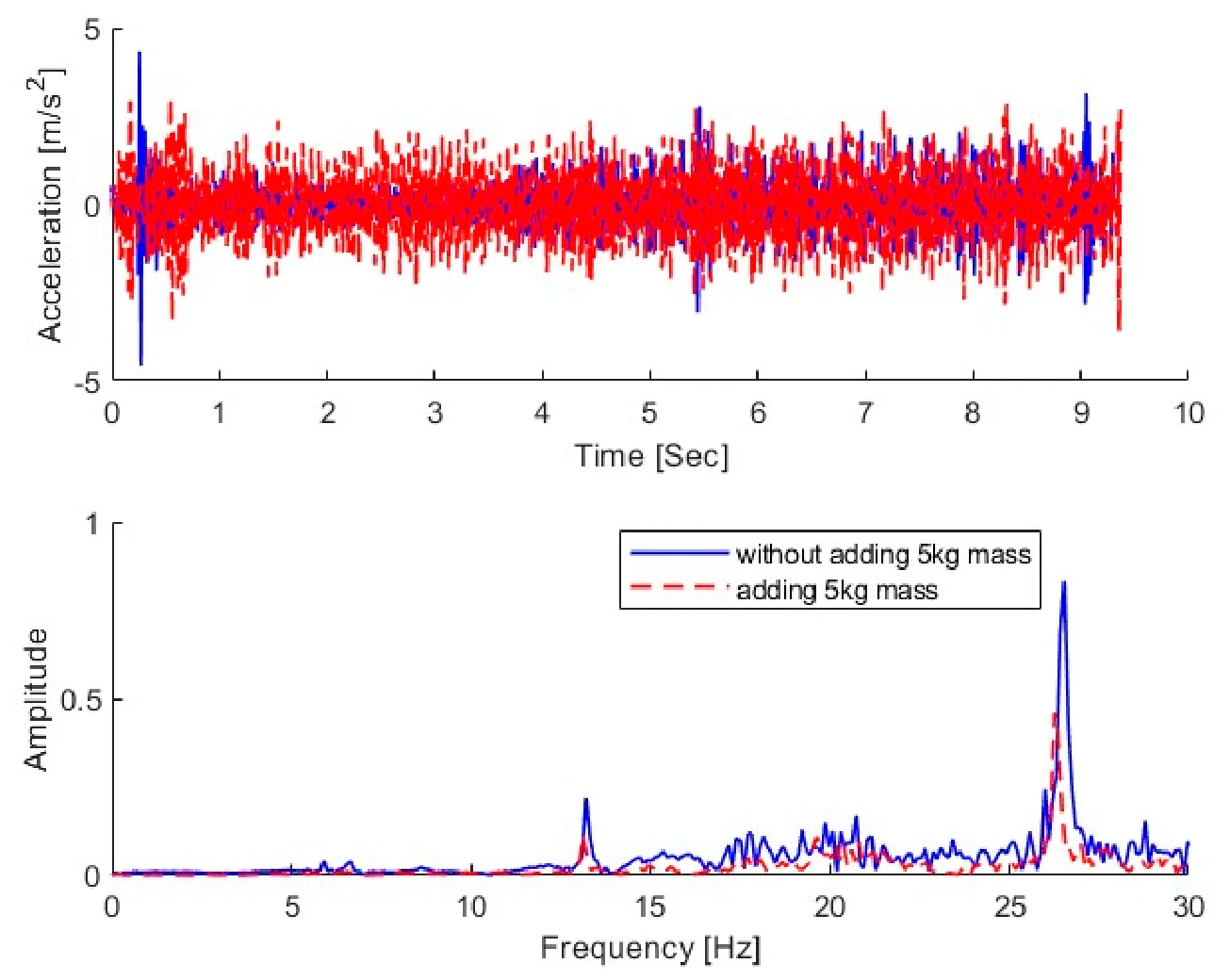
Dynamic responses and their Fourier spectra of the vehicle with 4 kg weight block.

**Figure 19:**
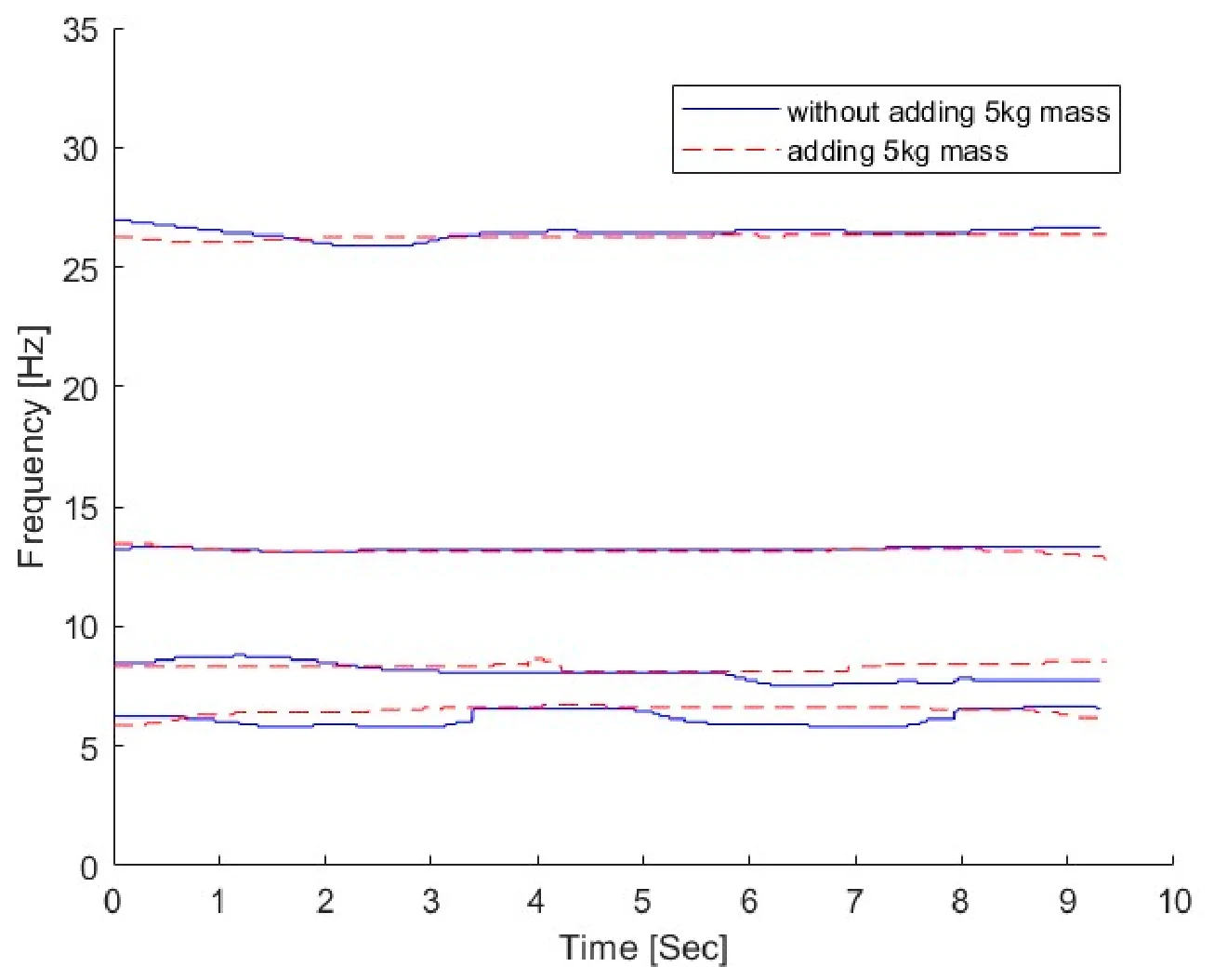
IFs from vehicle responses with 4 kg weight block.

In this study, a drive-by bridge time-varying feature extraction method is proposed to extract time-varying characteristics of vehicle–bridge interaction systems from dynamic responses of a passing vehicle. The bridge dynamic components are captured through pre-installed sensors on a moving vehicle passage over it. Numerical simulations in Section 3 have shown the significant influence of the vehicle–bridge coupling effect on the bridge instantaneous frequency extraction from the vehicle responses. Two key parameters for vehicle–bridge coupling effect, e.g., the vehicle–bridge mass ratio and the vehicle–bridge frequency ratio have been discussed. In this experimental study, a 4 kg weight block is added on the vehicle. Two cases with different vehicle–bridge mass ratios have been studied, e.g., without or with 4 kg weight block on the vehicle. The corresponding vehicle–bridge mass ratios are 0.069 and 0.126 respectively. Figure 16 and Figure 17 show dynamic response and its spectrum of the passing vehicle without 4 kg weight block, as well as IFs. Figure 18 and Figure 19 show dynamic response and its spectrum of the passing vehicle with 4 kg weight block, as well as IFs.

#### 5.2.1. The Vehicle Without 4 kg Weight Block

In this study, the average response measurements at the front and rear vehicle are used as the vehicle responses. Figure 16 shows dynamic responses of the vehicle passing over the main beam with or without the additional mass suspended at the midspan of the first span and their corresponding Fourier spectra. From the laser sensors, the average speed of the vehicle travelling over the main beam is 0.62 m/s. Figure 17 shows IFs extracted from the vehicle responses using the proposed SST2 method. From Figure 16, there are two peaks around 13 Hz and 27 Hz in the Fourier spectra. According to the proposed SST2 method, two IFs are extracted from the vertical response of the vehicle passing over the main beam without or with the additional 5 kg mass. These two IFs around the third and fourth natural frequencies of the bridge respectively. According to Figure 16 and Figure 17, there are no frequency components around the first two natural frequencies extracted from the vehicle responses. The first IF is around the third natural frequency of the bridge—13.34 Hz—and the second IF is above the fourth natural frequency of the bridge—24.45 Hz. According to Figure 17, the IFs with the additional 5 kg mass are slightly smaller than the corresponding IFs without the additional mass, especially around the location of the additional mass. This is due to the fact that additional mass reduces the bridge frequency. This further confirms that the change in IF is effective to indicate the damage location.

#### 5.2.2. The Vehicle with 4 kg Weight Block

To further study the influence of the vehicle–bridge coupling effect, a 4 kg mass block is added on the top of the vehicle. The vehicle–bridge mass ratio is about 0.126. Figure 18 shows dynamic responses of the vehicle crossing the bridge without or with 5 kg of additional mass and their corresponding spectra. From Figure 18, in addition to those two peaks around 13 Hz and 27 Hz, there are small peaks around 6 Hz and 8 Hz. Figure 19 shows the IFs extracted from dynamic responses of the passing vehicle. There are four IFs around four bridge natural frequencies in the figure. The results show that the increase in the vehicle–bridge mass ratio enables the vehicle–bridge coupling effect, and the low bridge mode information is captured by the passing instrumented vehicle.

As highlighted above, the vehicle–bridge mass ratio has a significant effect to capture the bridge dynamic components. The heavy vehicle could enhance the vehicle–bridge coupling effect. To extract the low bridge frequency components, a heavy vehicle with a high vehicle–bridge mass ratio is recommended.

## 6. Conclusions

A new drive-by bridge time-varying feature extraction method for bridge damage detection has been developed in this study. Numerical and experimental studies have been used to verify the performance of the proposed method to extract time-varying characteristics of VBI systems. The results show that extracted instantaneous frequencies are sensitive to simulated damage or structural change. Some conclusions are obtained as follows:

(1) Compared to the traditional SST, SST2 provides clearer and more accurate TFR when dealing with complex signals, especially for the VBI systems.
(2) The effect of vehicle parameters on the time-varying characteristics of the VBI system has been studied. Numerical results show that the proposed method is much more robust to the measurement noise. The vehicle–bridge mass ratio and the vehicle–bridge frequency ratio have a significant effect on time-varying characteristics of the VBI systems. To get the accurate TFR, it requires a low vehicle speed.
(3) Different damage scenarios have been simulated, and the corresponding time-varying characteristics of the VBI systems are extracted using the proposed method. Numerical results show that patterns of TFRs and IFs are significantly affected by the damage severity and location of the bridge. The time-varying features from dynamic responses of the vehicle passing over the bridge could be used to indicate the bridge damage.
(4) The proposed drive-by time-varying feature extraction method is verified using a single distributed damage zone scenario, which frequently occurs in a localised area experienced high stress, material degradation or cracking of concrete bridges. The single damage zone is simulated by a localised stiffness reduction in numerical simulation and a structural change through adding mass in experimental study. Further study is needed to extend the proposed method for other damage scenarios, such as multiple damage locations, support deterioration and expansion joint damage.
(5) Numerical simulations and laboratory experiments have shown the great potential of the proposed drive-by bridge time-varying feature extraction for condition assessment of the bridge in practice. For complex vehicle–bridge interaction systems, further laboratory tests with stiffness reduction and field tests are needed to verify the proposed method for practical applications. Future research involves machine learning based bridge damage detection using time-varying characteristics of VBI systems.

## Figures and Tables

**Table 1:** Parameters of different vehicles.

Case	Stiffness (N/m)	Vehicle Mass (kg)	Frequency of Vehicle (Hz)	Vehicle/Bridge Mass Ratio	Vehicle/Bridge Frequency Ratio
1	3.53 × 10^5^	875	3.20	0.0048	0.90
2	7.05 × 10^5^	1750	3.20	0.0095	0.90
3	1.41 × 10^6^	3500	3.20	0.0190	0.90
4	2.82 × 10^6^	7000	3.20	0.0390	0.90
5	1.71 × 10^6^	7000	2.49	0.0390	0.70
6	4.23 × 10^6^	7000	3.91	0.0390	1.10
7	1.01 × 10^7^	7000	6.04	0.0390	1.70

**Table 2:** MSE values for different roughness, vehicle speed and measurement noise.

Parameters	Road Roughness			Vehicle Speed (m/s)			Measurement Noise (%)		
	A	B	C	1	2	4	0	5	10
Scenarios	1	2	3	4	5	6	7	8	9
MSE	-	0.0028	0.0028	-	0.0036	0.0129	-	0.0000	0.0000

Note: ‘-’ is the reference baseline.

**Table 3:** MSE values for different damage scenarios.

Parameters	Damage Severity (α)			Damage Region Parameter (β)			Damage Location (*l_c_*/*L*)		
	0.0	0.3	0.5	0.00	0.10	0.25	0.125	0.250	0.50
Scenarios	10	11	12	13	14	15	16	17	18
MSE	-	0.0081	0.0187	-	0.0081	0.0177	0.0645	0.0188	-

Note: ‘-’ is the reference.

## Data Availability

The data presented in this study are available on request from the corresponding author.
